# Supplementary material for: Education shapes the link between EEG aperiodic components and cognitive aging
Source: PLoS One. 2026 Mar 9;21(3):e0328318. doi: 10.1371/journal.pone.0328318 (PMC12970889; doi:10.1371/journal.pone.0328318)
Supplement: S1 File — Table S1. Significance of the smooth terms (interactions and main effects of age and education) for the exponent models; Table S2. Significance of the smooth terms (interactions and main effects of age and education) for the offset models; Table S3. Significance of the smooth terms (interactions and main effects of age and education) for the baseline MMSE model; Table S4. Significance of the smooth terms (interactions and main effects of age, education, and exponent) for the MMSE and exponent models; Table S5. Significance of the smooth terms (interactions and main effects of age, education, and offset) for the MMSE and offset models; Table S6. Spearman’s correlation coefficients between exponent and offset for each ROI; Figure S1. Distribution of education years by age. The distribution of education levels is uniform across age; Figure S2. Main effect of age on exponent for the simple models of right cingulate, left and right occipital, right parietal, and right temporal; Figure S3. Main effect of education on exponent for the simple models of right cingulate, bilateral occipital, right parietal and right temporal; Figure S4. Main effect of age on offset for the simple models of right cingulate, bilateral occipital, bilateral parietal and bilateral temporal; Figure S5. Main effect of education on offset for the simple models of right cingulate, bilateral occipital, bilateral parietal and bilateral temporal; Figure S6. MMSE score changes according to age and education across different exponent levels for the bilateral cingulate; Figure S7. MMSE score changes according to age and education across different exponent levels for the bilateral hippocampus; Figure S8. MMSE score changes according to age and education across different exponent levels for the bilateral occipital regions; Figure S9. MMSE score changes according to age and education across different exponent levels for the bilateral parietal regions; Figure S10. Comparisons and significant differences between [file pone.0328318.s001.docx]

# Methods


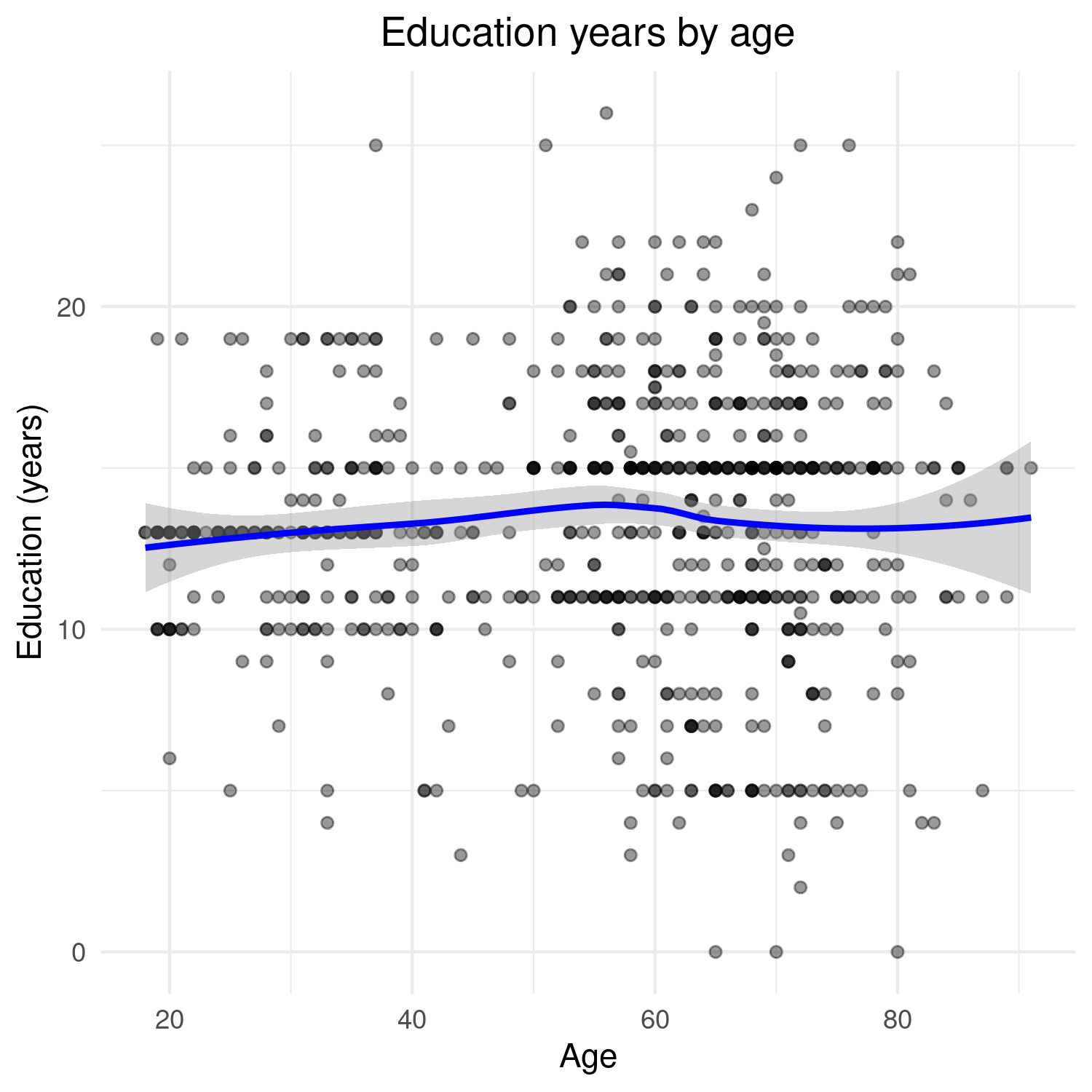


Figure S1. Distribution of education years by age. The distribution of education levels is reasonably uniform and spread across age range.

# Aim 1: EEG components, age and education

## Exponent

### Significance of the smooth terms

Table S1. Significance of the smooth terms (interactions and main effects of age and education) for the exponent models.

|  | ROI | type | deviance | EDF | F | p value | Sig |
| --- | --- | --- | --- | --- | --- | --- | --- |
| s(Age) | CING_left | complex | 5.797 | 3.009 | 6.419 | <0.001 | * |
| s(Educ) | CING_left | complex | 5.797 | 2.416 | 814 | 0.518 |  |
| ti(Age,Educ) | CING_left | complex | 5.797 | 1.004 | 5.155 | 0.023 | * |
| s(Age) | HPC_left | complex | 17.098 | 4.37 | 11.94 | <0.001 | * |
| s(Educ) | HPC_left | complex | 17.098 | 5.903 | 2.881 | 0.006 | * |
| ti(Age,Educ) | HPC_left | complex | 17.098 | 3.108 | 3.272 | 0.013 | * |
| s(Age) | HPC_right | complex | 15.426 | 2.929 | 17.201 | <0.001 | * |
| s(Educ) | HPC_right | complex | 15.426 | 6.599 | 2.796 | 0.004 | * |
| ti(Age,Educ) | HPC_right | complex | 15.426 | 1.684 | 2.591 | 0.069 |  |
| s(Age) | PARIET_left | complex | 12.945 | 3.589 | 11.984 | <0.001 | * |
| s(Educ) | PARIET_left | complex | 12.945 | 5.586 | 1.872 | 0.082 |  |
| ti(Age,Educ) | PARIET_left | complex | 12.945 | 1.001 | 2.928 | 0.087 |  |
| s(Age) | TEMP_left | complex | 22.11 | 5.909 | 12.121 | <0.001 | * |
| s(Educ) | TEMP_left | complex | 22.11 | 6.641 | 3.179 | 0.002 | * |
| ti(Age,Educ) | TEMP_left | complex | 22.11 | 3.317 | 3.023 | 0.025 | * |
| s(Age) | CING_right | simple | 6.75 | 2.481 | 8.608 | <0.001 | * |
| s(Educ) | CING_right | simple | 6.75 | 4.208 | 2.111 | 0.056 |  |
| s(Age) | OCC_left | simple | 10.071 | 3.533 | 12.701 | <0.001 | * |
| s(Educ) | OCC_left | simple | 10.071 | 4.007 | 1.221 | 0.29 |  |
| s(Age) | OCC_right | simple | 10.27 | 2.998 | 20.153 | <0.001 | * |
| s(Educ) | OCC_right | simple | 10.27 | 1.002 | 0.05 | 0.827 |  |
| s(Age) | PARIET_right | simple | 7.962 | 2.76 | 12.964 | <0.001 | * |
| s(Educ) | PARIET_right | simple | 7.962 | 3.071 | 1.362 | 0.282 |  |
| s(Age) | TEMP_right | simple | 17.923 | 3.442 | 19.504 | <0.001 | * |
| s(Educ) | TEMP_right | simple | 17.923 | 6.584 | 3.026 | 0.006 | * |

### Simple models results

In right cingulate, left and right occipital, right parietal, and right temporal, the simple models were preferable over the complex ones.

In these areas, shapes of the main effects of age and education on exponent are fairly consistent across ROIs and show a decrease in exponent as individuals age. The decrease looks more marked between 20 and 60 years, while after 60 and towards the end of life the decrease in slope values is attenuated (Figure S2). This might indicate that our slope flattens faster between 20 and 60, and this decline slows down after 60.


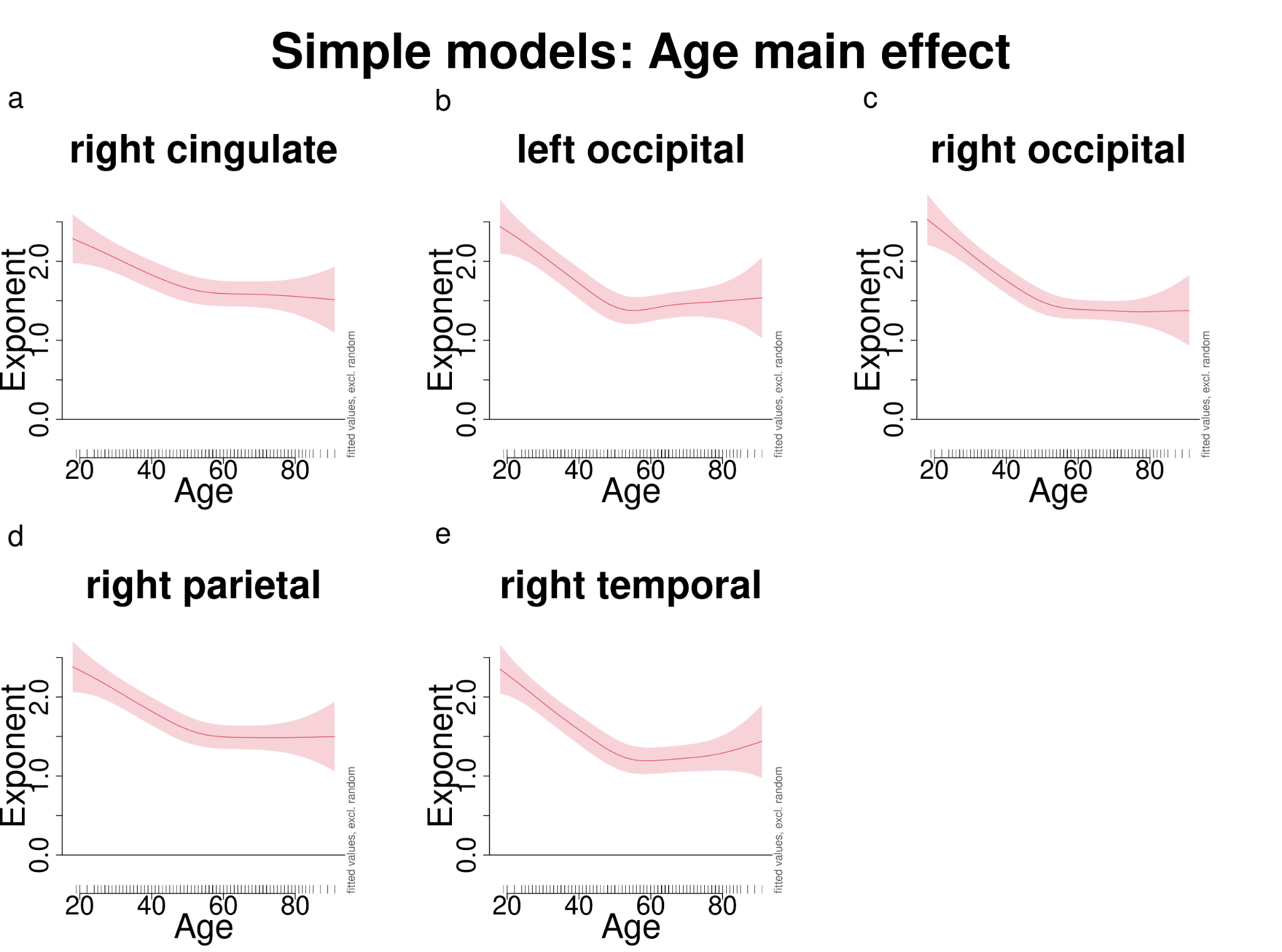


Figure S2. Main effect of age on exponent for the simple models of right cingulate, left and right occipital, right parietal, and right temporal.

Also the shapes of the main effect of education are fairly consistent across brain regions, except for the right occipital (Figure S3). Higher education levels are associated with slightly higher exponents (interpretable as higher neurocognitive efficiency).


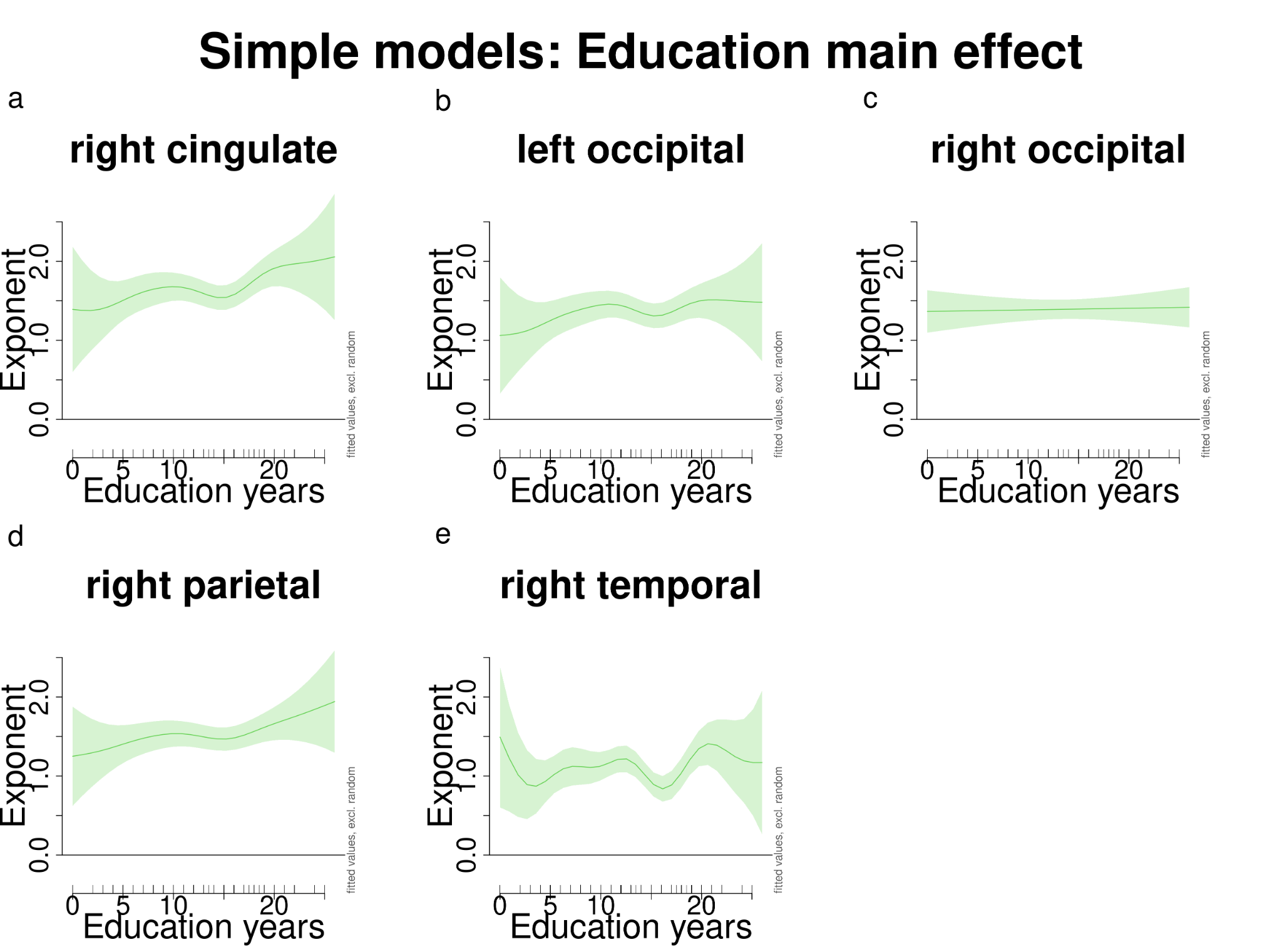


Figure S3. Main effect of education on exponent for the simple models of right cingulate, bilateral occipital, right parietal and right temporal.

## Offset

### Significance of the smooth terms

Table S2. Significance of the smooth terms (interactions and main effects of age and education) for the offset models.

|  | ROI | type | deviance | EDF | F | p value | Sig |
| --- | --- | --- | --- | --- | --- | --- | --- |
| s(Age) | CING_left | complex | 3.444 | 3.767 | 2.182 | 0.047 | * |
| s(Educ) | CING_left | complex | 3.444 | 1.805 | 307 | 0.668 |  |
| ti(Age,Educ) | CING_left | complex | 3.444 | 1.191 | 5.816 | 0.014 | * |
| s(Age) | HPC_left | complex | 8.354 | 4.264 | 8.645 | <0.001 | * |
| s(Educ) | HPC_left | complex | 8.354 | 2.027 | 335 | 0.667 |  |
| ti(Age,Educ) | HPC_left | complex | 8.354 | 1.001 | 6.426 | 0.011 | * |
| s(Age) | HPC_right | complex | 8.115 | 3.451 | 9.982 | <0.001 | * |
| s(Educ) | HPC_right | complex | 8.115 | 2.955 | 1.681 | 0.154 |  |
| ti(Age,Educ) | HPC_right | complex | 8.115 | 1.008 | 4.974 | 0.026 | * |
| s(Age) | CING_right | simple | 3.135 | 2.796 | 3.16 | 0.022 | * |
| s(Educ) | CING_right | simple | 3.135 | 3.158 | 1.555 | 0.182 |  |
| s(Age) | OCC_left | simple | 8.037 | 3.982 | 9.072 | <0.001 | * |
| s(Educ) | OCC_left | simple | 8.037 | 3.422 | 866 | 0.441 |  |
| s(Age) | OCC_right | simple | 8.589 | 3.087 | 15.318 | <0.001 | * |
| s(Educ) | OCC_right | simple | 8.589 | 1.913 | 141 | 0.809 |  |
| s(Age) | PARIET_left | simple | 9.423 | 3.723 | 10.264 | <0.001 | * |
| s(Educ) | PARIET_left | simple | 9.423 | 3.878 | 1.14 | 0.317 |  |
| s(Age) | PARIET_right | simple | 7.397 | 3.213 | 10.654 | <0.001 | * |
| s(Educ) | PARIET_right | simple | 7.397 | 3.035 | 1.095 | 0.399 |  |
| s(Age) | TEMP_left | simple | 11.021 | 4.911 | 11.977 | <0.001 | * |
| s(Educ) | TEMP_left | simple | 11.021 | 1.002 | 1 | 0.989 |  |
| s(Age) | TEMP_right | simple | 11.894 | 4.419 | 11.22 | <0.001 | * |
| s(Educ) | TEMP_right | simple | 11.894 | 4.741 | 1.761 | 0.158 |  |

### Simple models results

In right cingulate, bilateral occipital, bilateral parietal and bilateral temporal, the simple models were preferable over the complex ones.

In these areas, shapes of the main effects of age and education on offset are fairly consistent across ROIs and show a decrease in exponent as individuals age. The decrease looks more marked between 20 and 60 years, while after 60 and towards the end of life the decrease in offset values is attenuated (Figure S4). This might indicate that the offset decreases faster between 20 and 60, and this decline slows down after 60 (mirroring results for slope).


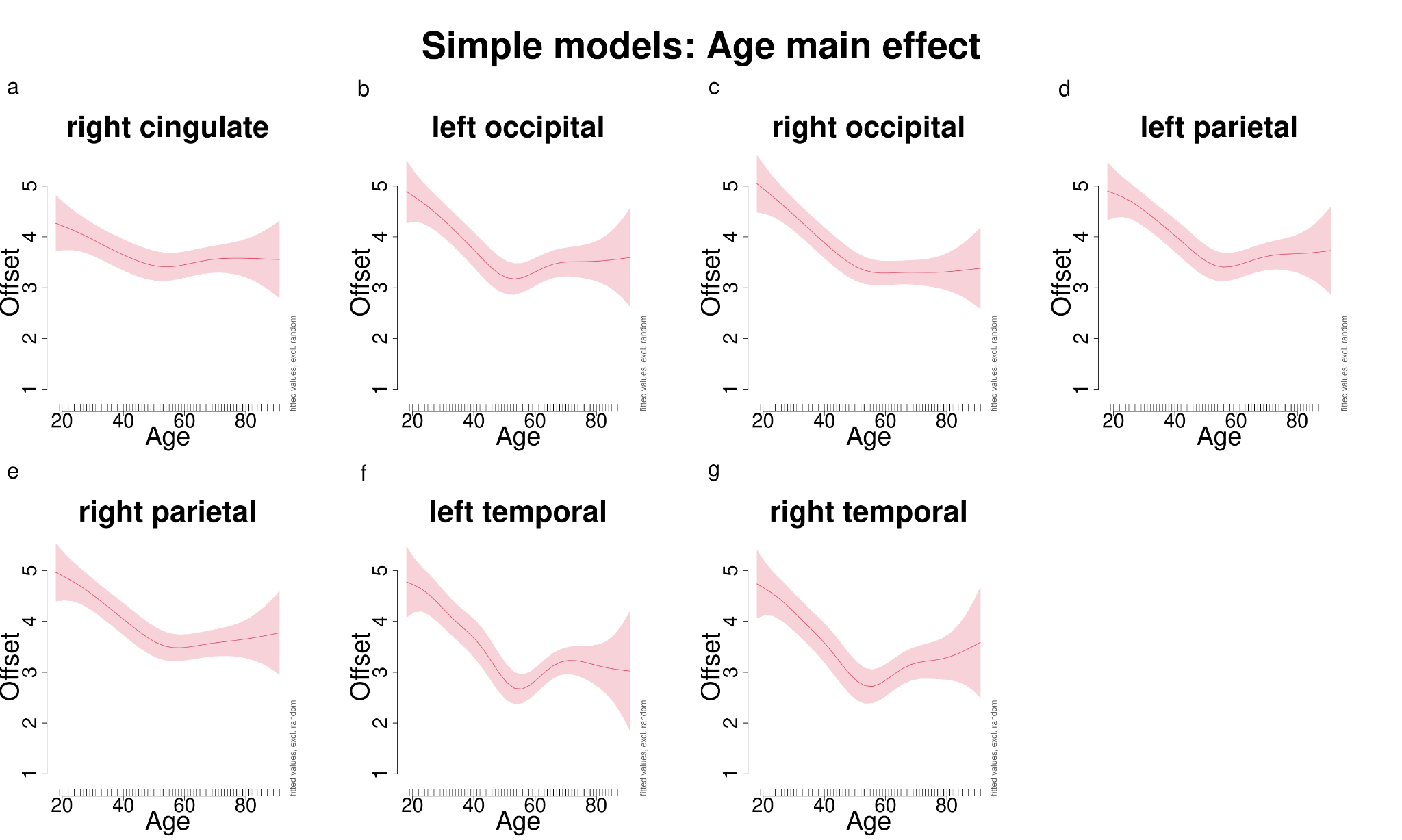


Figure S4. Main effect of age on offset for the simple models of right cingulate, bilateral occipital, bilateral parietal and bilateral temporal.


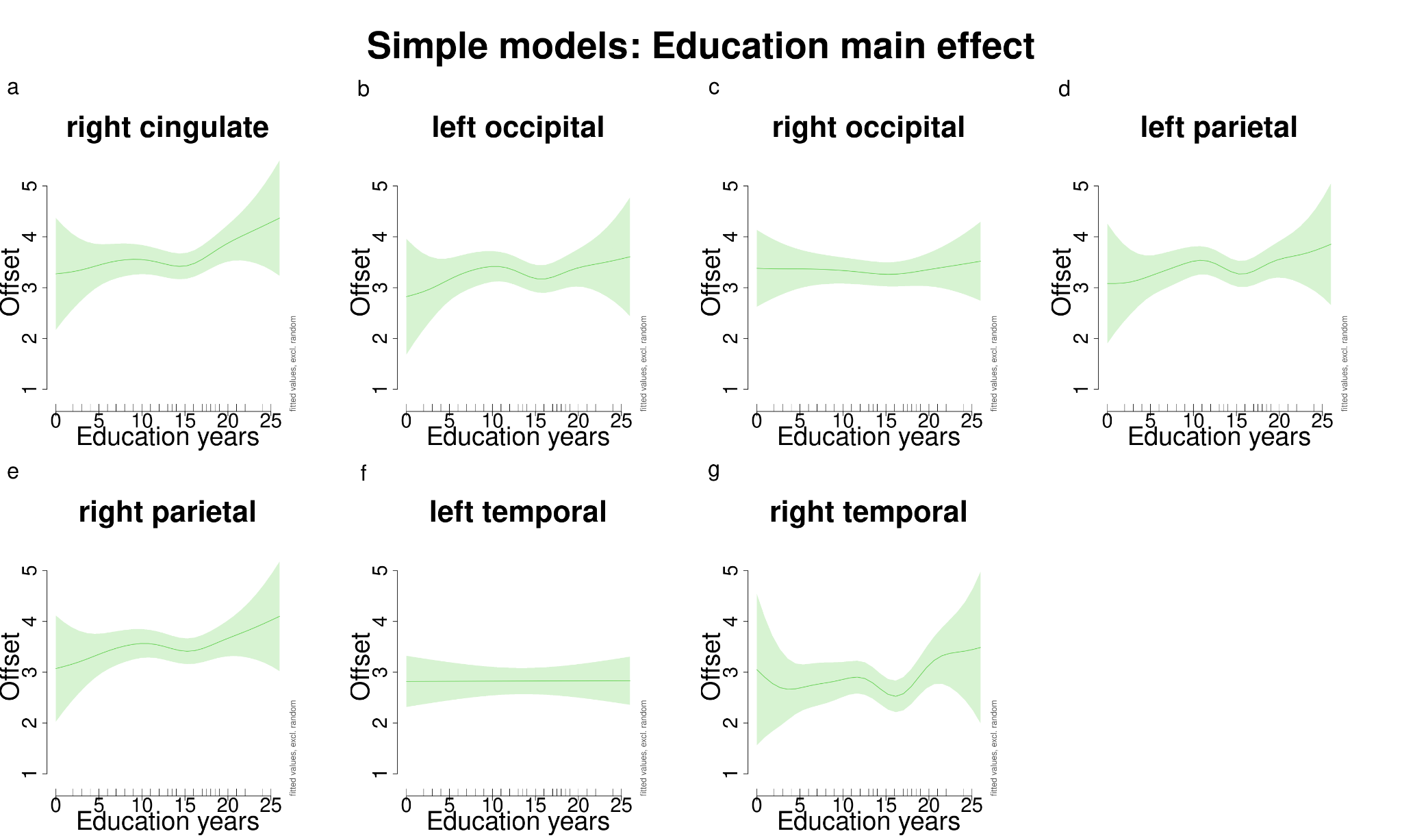


Figure S5. Main effect of education on offset for the simple models of right cingulate, bilateral occipital, bilateral parietal and bilateral temporal.

Also the shapes of the main effect of education are fairly consistent across brain regions, except for the right occipital and left temporal (Figure S5). Higher education levels are associated with slightly higher offsets.

# Aim 2: changes in cognition and EEG components across age and education

## MMSE

### Significance of the smooth terms

Table S3. Significance of the smooth terms (interactions and main effects of age and education) for the baseline MMSE model.

|  | deviance | EDF | F | p value | Sig |
| --- | --- | --- | --- | --- | --- |
| s(Age) | 18.957 | 5.412 | 9.244 | <0.001 | * |
| s(Educ) | 18.957 | 2.808 | 14.038 | <0.001 | * |
| ti(Age,Educ) | 18.957 | 1.469 | 0.173 | 0.778 |  |

##

## MMSE and Exponent

### Significance of the smooth terms

Table S4. Significance of the smooth terms (interactions and main effects of age, education and exponent) for the MMSE and exponent models.

|  | ROI | deviance | EDF | F | p value | Sig |
| --- | --- | --- | --- | --- | --- | --- |
| s(Age) | CING_left | 21.356 | 3.892 | 11.691 | <0.001 | * |
| s(Educ) | CING_left | 21.356 | 2.212 | 10.764 | <0.001 | * |
| s(slope) | CING_left | 21.356 | 2.049 | 6.925 | <0.001 | * |
| ti(Age,Educ) | CING_left | 21.356 | 1 | 286 | 0.593 |  |
| ti(Age,slope) | CING_left | 21.356 | 2.45 | 1.554 | 0.139 |  |
| ti(Age,Educ,slope) | CING_left | 21.356 | 1.462 | 5.021 | 0.02 | * |
| s(Age) | CING_right | 21.226 | 4.008 | 8.652 | <0.001 | * |
| s(Educ) | CING_right | 21.226 | 2.39 | 9.404 | <0.001 | * |
| s(slope) | CING_right | 21.226 | 2.439 | 5.883 | 0.001 | * |
| ti(Age,Educ) | CING_right | 21.226 | 1.321 | 422 | 0.725 |  |
| ti(Age,slope) | CING_right | 21.226 | 2.189 | 653 | 0.398 |  |
| ti(Age,Educ,slope) | CING_right | 21.226 | 2.323 | 0.97 | 0.395 |  |
| s(Age) | HPC_left | 20.942 | 3.988 | 8.839 | <0.001 | * |
| s(Educ) | HPC_left | 20.942 | 1 | 22.624 | <0.001 | * |
| s(slope) | HPC_left | 20.942 | 1 | 4.824 | 0.028 | * |
| ti(Age,Educ) | HPC_left | 20.942 | 2.024 | 1.075 | 0.462 |  |
| ti(Age,slope) | HPC_left | 20.942 | 3.957 | 2.6 | 0.025 | * |
| ti(Age,Educ,slope) | HPC_left | 20.942 | 1 | 6.088 | 0.014 | * |
| s(Age) | HPC_right | 20.428 | 4.388 | 8.658 | <0.001 | * |
| s(Educ) | HPC_right | 20.428 | 1 | 26.577 | <0.001 | * |
| s(slope) | HPC_right | 20.428 | 1 | 3.746 | 0.053 |  |
| ti(Age,Educ) | HPC_right | 20.428 | 2.093 | 1.613 | 0.261 |  |
| ti(Age,slope) | HPC_right | 20.428 | 4.768 | 1.704 | 0.116 |  |
| ti(Age,Educ,slope) | HPC_right | 20.428 | 1 | 2.989 | 0.084 |  |
| s(Age) | OCC_left | 20.733 | 4 | 8.235 | <0.001 | * |
| s(Educ) | OCC_left | 20.733 | 1.955 | 11.538 | <0.001 | * |
| s(slope) | OCC_left | 20.733 | 1.653 | 4.648 | 0.009 | * |
| ti(Age,Educ) | OCC_left | 20.733 | 1.532 | 724 | 0.514 |  |
| ti(Age,slope) | OCC_left | 20.733 | 2.418 | 866 | 0.34 |  |
| ti(Age,Educ,slope) | OCC_left | 20.733 | 2.229 | 3.229 | 0.022 | * |
| s(Age) | OCC_right | 20.526 | 4.339 | 7.348 | <0.001 | * |
| s(Educ) | OCC_right | 20.526 | 2.184 | 10.024 | <0.001 | * |
| s(slope) | OCC_right | 20.526 | 1.689 | 4.953 | 0.006 | * |
| ti(Age,Educ) | OCC_right | 20.526 | 1.619 | 692 | 0.496 |  |
| ti(Age,slope) | OCC_right | 20.526 | 2.456 | 1.791 | 0.098 |  |
| ti(Age,Educ,slope) | OCC_right | 20.526 | 1 | 5.172 | 0.023 | * |
| s(Age) | PARIET_left | 20.244 | 3.926 | 10.269 | <0.001 | * |
| s(Educ) | PARIET_left | 20.244 | 2.157 | 9.257 | <0.001 | * |
| s(slope) | PARIET_left | 20.244 | 1.242 | 8.052 | 0.001 | * |
| ti(Age,Educ) | PARIET_left | 20.244 | 1 | 461 | 0.497 |  |
| ti(Age,slope) | PARIET_left | 20.244 | 2.038 | 851 | 0.336 |  |
| ti(Age,Educ,slope) | PARIET_left | 20.244 | 1.855 | 3.433 | 0.022 | * |
| s(Age) | PARIET_right | 19.978 | 4.044 | 8.711 | <0.001 | * |
| s(Educ) | PARIET_right | 19.978 | 2.643 | 8.073 | <0.001 | * |
| s(slope) | PARIET_right | 19.978 | 2.017 | 6.573 | 0.001 | * |
| ti(Age,Educ) | PARIET_right | 19.978 | 1.197 | 417 | 0.713 |  |
| ti(Age,slope) | PARIET_right | 19.978 | 1.708 | 596 | 0.512 |  |
| ti(Age,Educ,slope) | PARIET_right | 19.978 | 1 | 3.594 | 0.058 |  |
| s(Age) | TEMP_left | 21.235 | 3.706 | 9.45 | <0.001 | * |
| s(Educ) | TEMP_left | 21.235 | 1 | 24.111 | <0.001 | * |
| s(slope) | TEMP_left | 21.235 | 1.397 | 1.301 | 0.189 |  |
| ti(Age,Educ) | TEMP_left | 21.235 | 2.027 | 1.543 | 0.318 |  |
| ti(Age,slope) | TEMP_left | 21.235 | 4.876 | 1.868 | 0.068 |  |
| ti(Age,Educ,slope) | TEMP_left | 21.235 | 1.828 | 3.03 | 0.036 | * |
| s(Age) | TEMP_right | 20.187 | 4.266 | 8.451 | <0.001 | * |
| s(Educ) | TEMP_right | 20.187 | 1 | 26.581 | <0.001 | * |
| s(slope) | TEMP_right | 20.187 | 1 | 4.369 | 0.037 | * |
| ti(Age,Educ) | TEMP_right | 20.187 | 1.885 | 1.343 | 0.348 |  |
| ti(Age,slope) | TEMP_right | 20.187 | 5.335 | 1.153 | 0.327 |  |
| ti(Age,Educ,slope) | TEMP_right | 20.187 | 1 | 2.285 | 0.131 |  |

The complex models showed a better fit than the baseline MMSE model in all the ROIs.

### Complex models’ figures

Figures S6-S9 depict MMSE score changes according to age and education across different exponent levels (results of the complex models’ interactions) for the bilateral cingulate, hippocampus, occipital and parietal ROIs. Darker blue shades indicate lower MMSE scores, while lighter green and yellow shades indicate higher MMSE scores. White areas indicate model estimates exceeding the upper MMSE score limit of 30.

Figures S10-S13 depict the comparisons and significant differences between MMSE scores of participants with high and low exponent, in relation with age and different education levels for the left hippocampuss, left temporal, right occipital and bilateral parietal ROIs. Upper panels show how different exponent levels shape MMSE scores across different levels of education (high and low) and increasing age. Lower panels show significant differences in MMSE scores between participants with varying exponent and education levels across age. In general, participants with low education and higher exponents have significantly higher MMSE scores than those with lower exponents starting approximately from age 40. Moreover, participants with high education and higher exponents have worse MMSE scores than those with lower exponents. In lower panels, age ranges when differences are significant are highlighted with a red line on the x axis (MMSE score differences are significant across the entire age window).


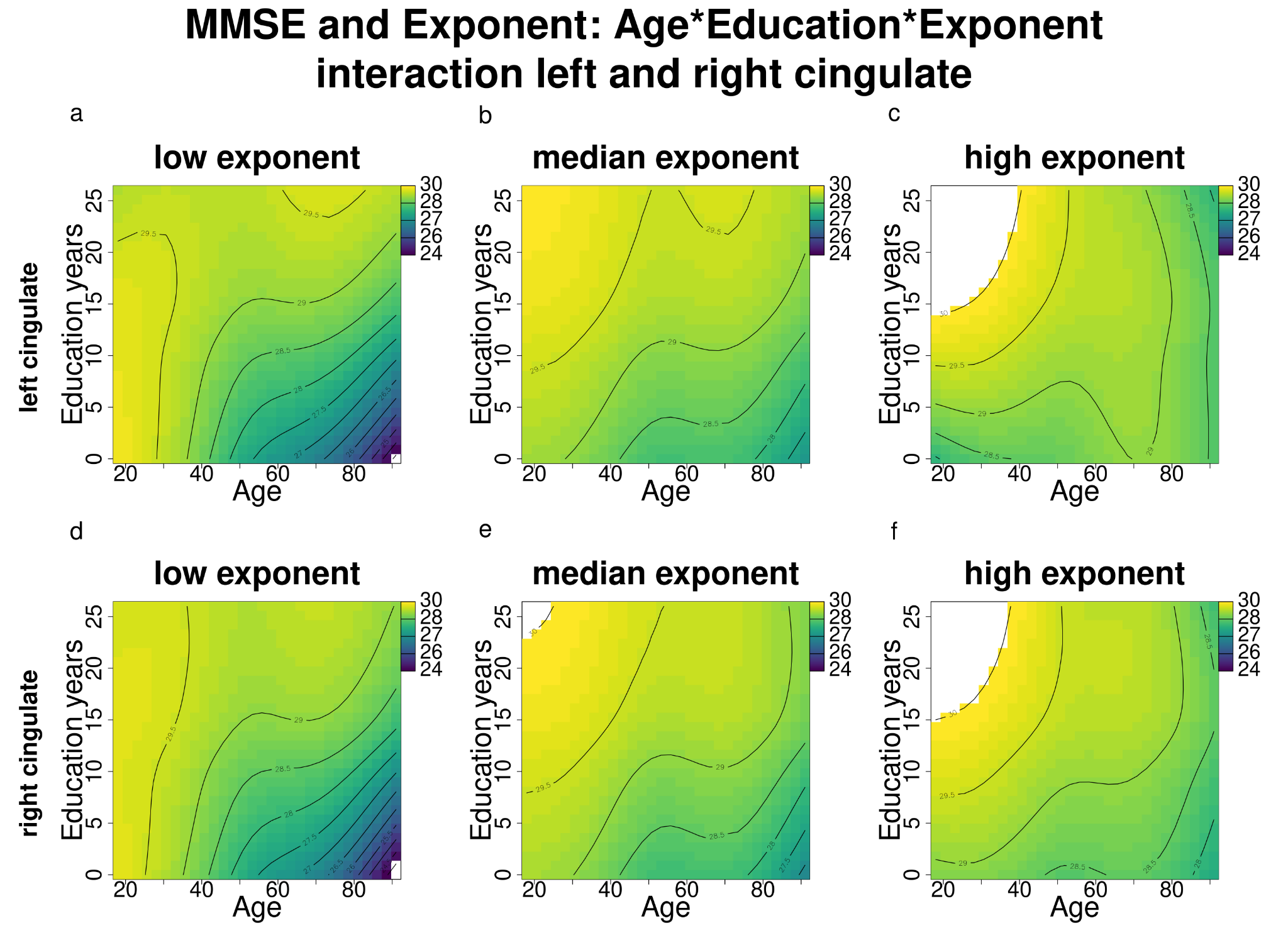


Figure S6. MMSE score changes according to age and education across different exponent levels for the bilateral cingulate.


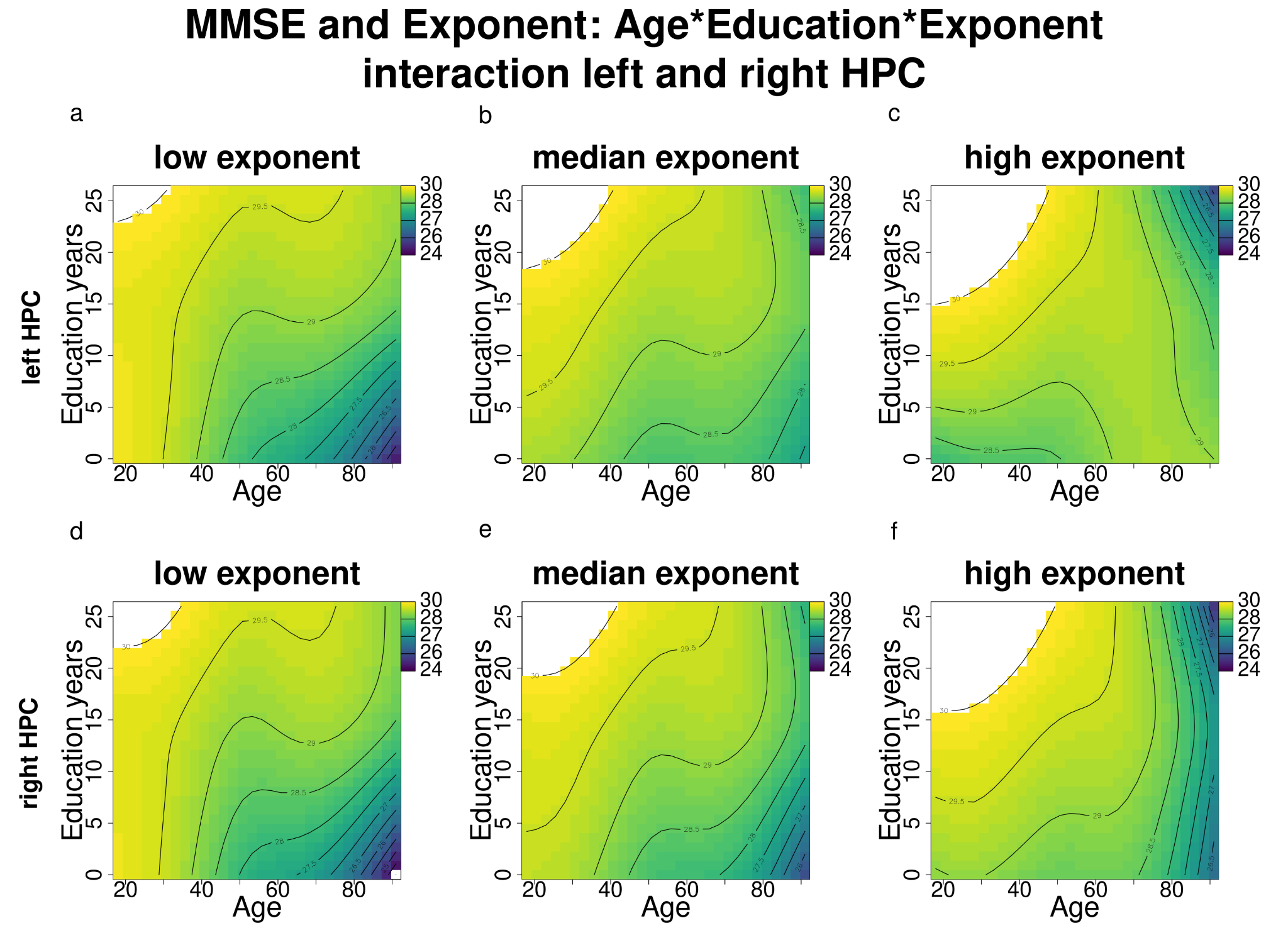


Figure S7. MMSE score changes according to age and education across different exponent levels for the bilateral hippocampus.


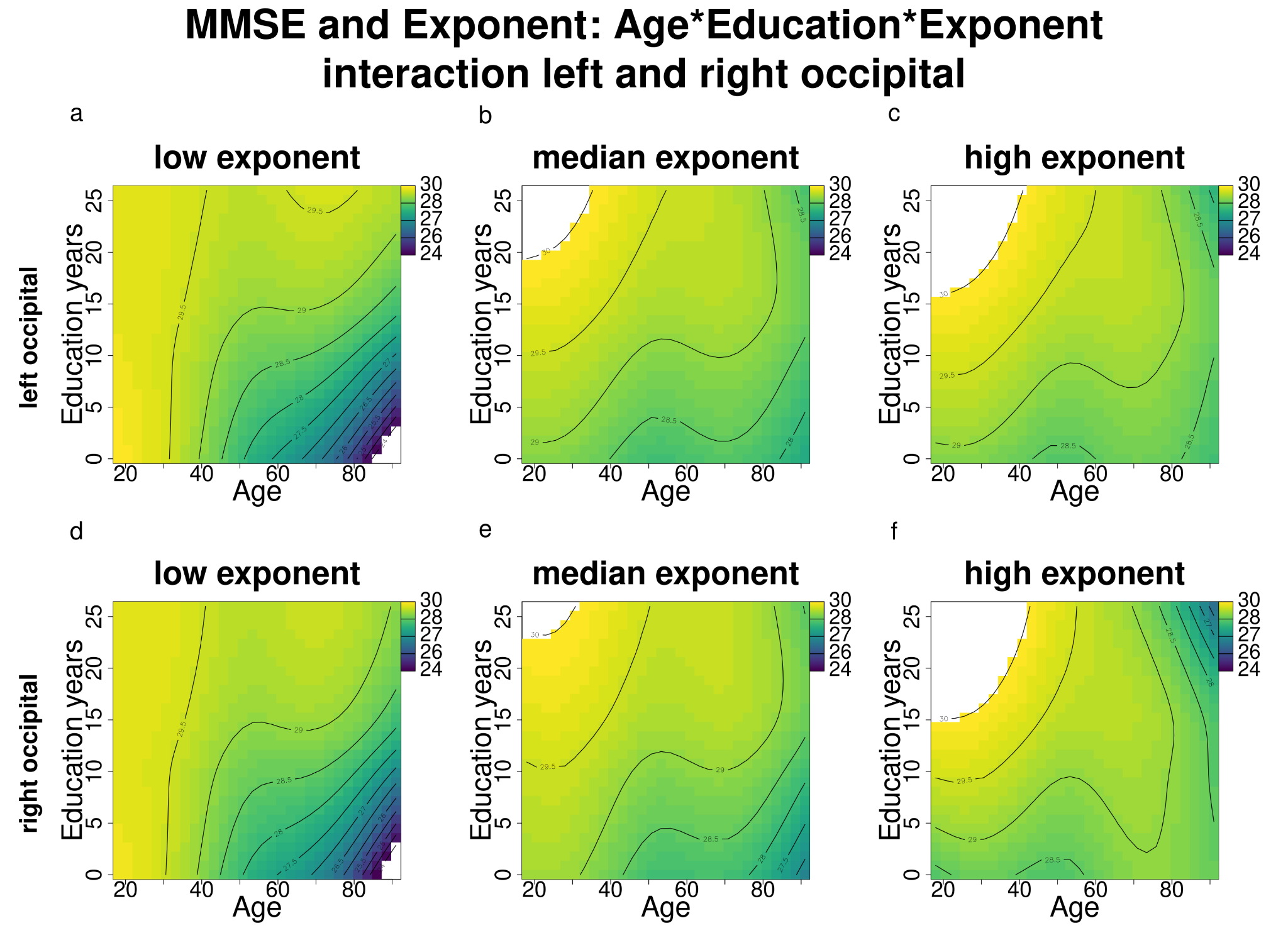


Figure S8. MMSE score changes according to age and education across different exponent levels for the bilateral occipital regions.


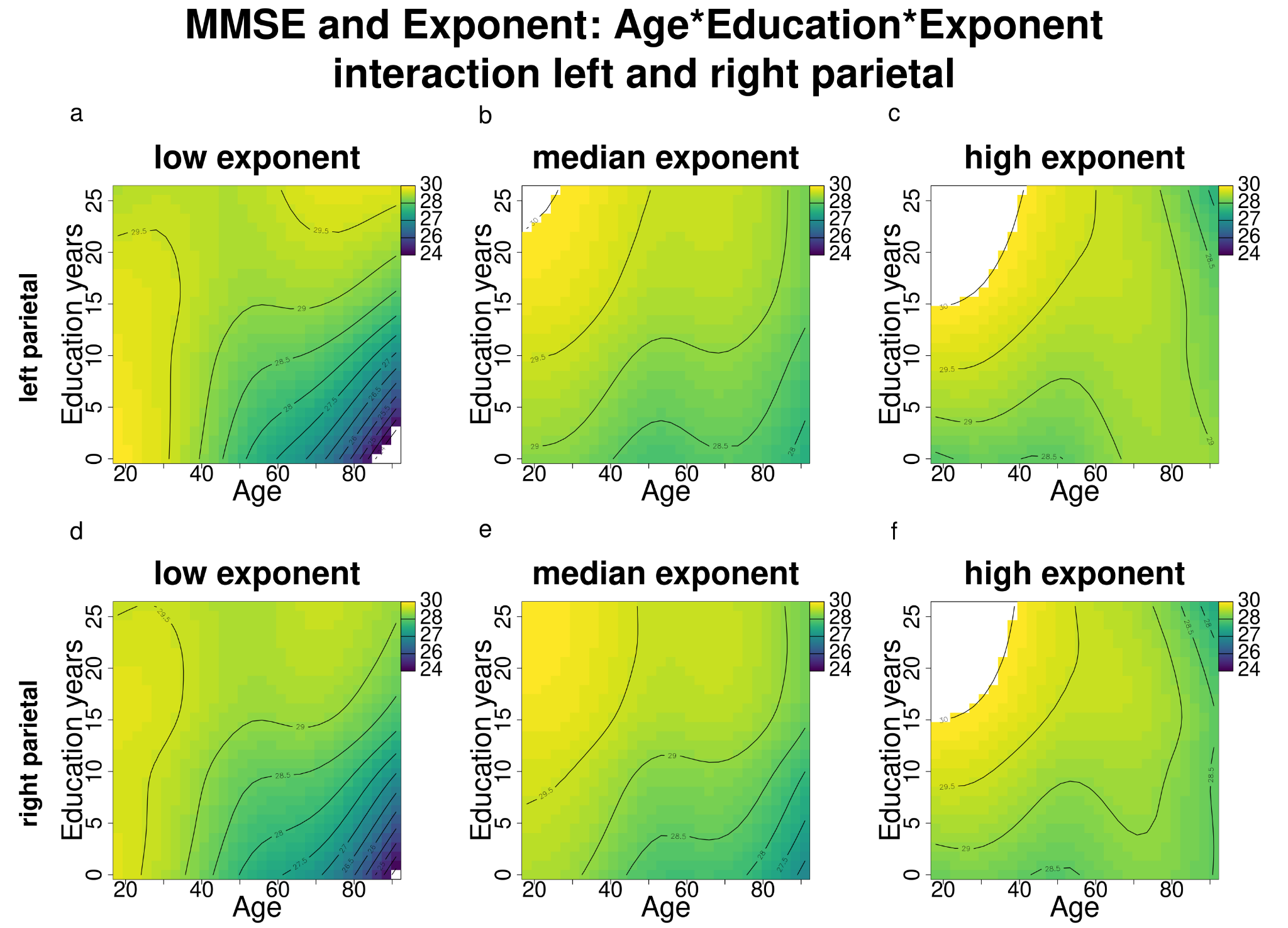


Figure S9. MMSE score changes according to age and education across different exponent levels for the bilateral parietal regions.


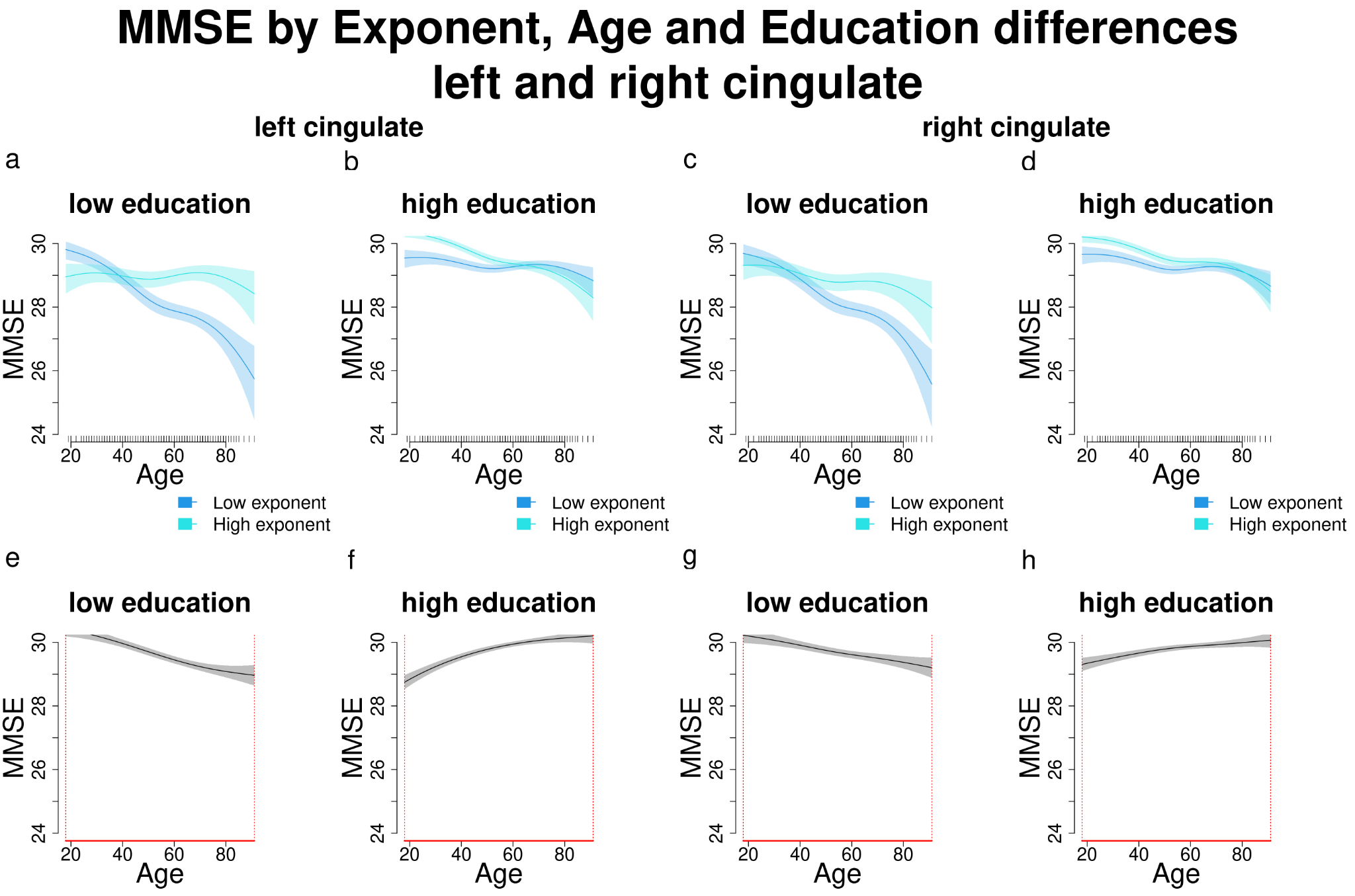


Figure S10. Comparisons and significant differences between MMSE scores of participants with high and low exponents, in relation with age and different education levels for the bilateral cingulate.


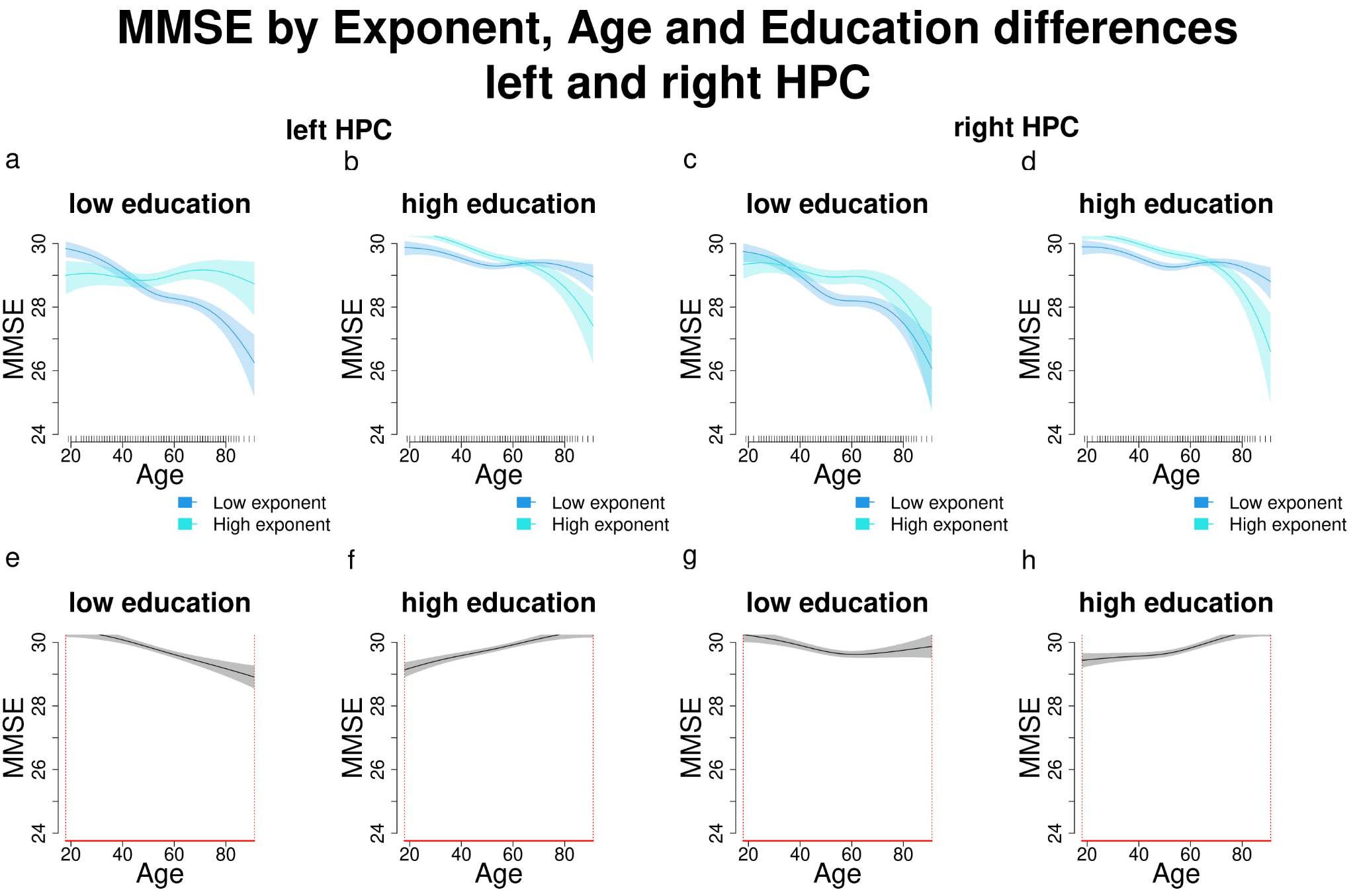


Figure S11. Comparisons and significant differences between MMSE scores of participants with high and low exponent, in relation with age and different education levels for the bilateral hippocampus.


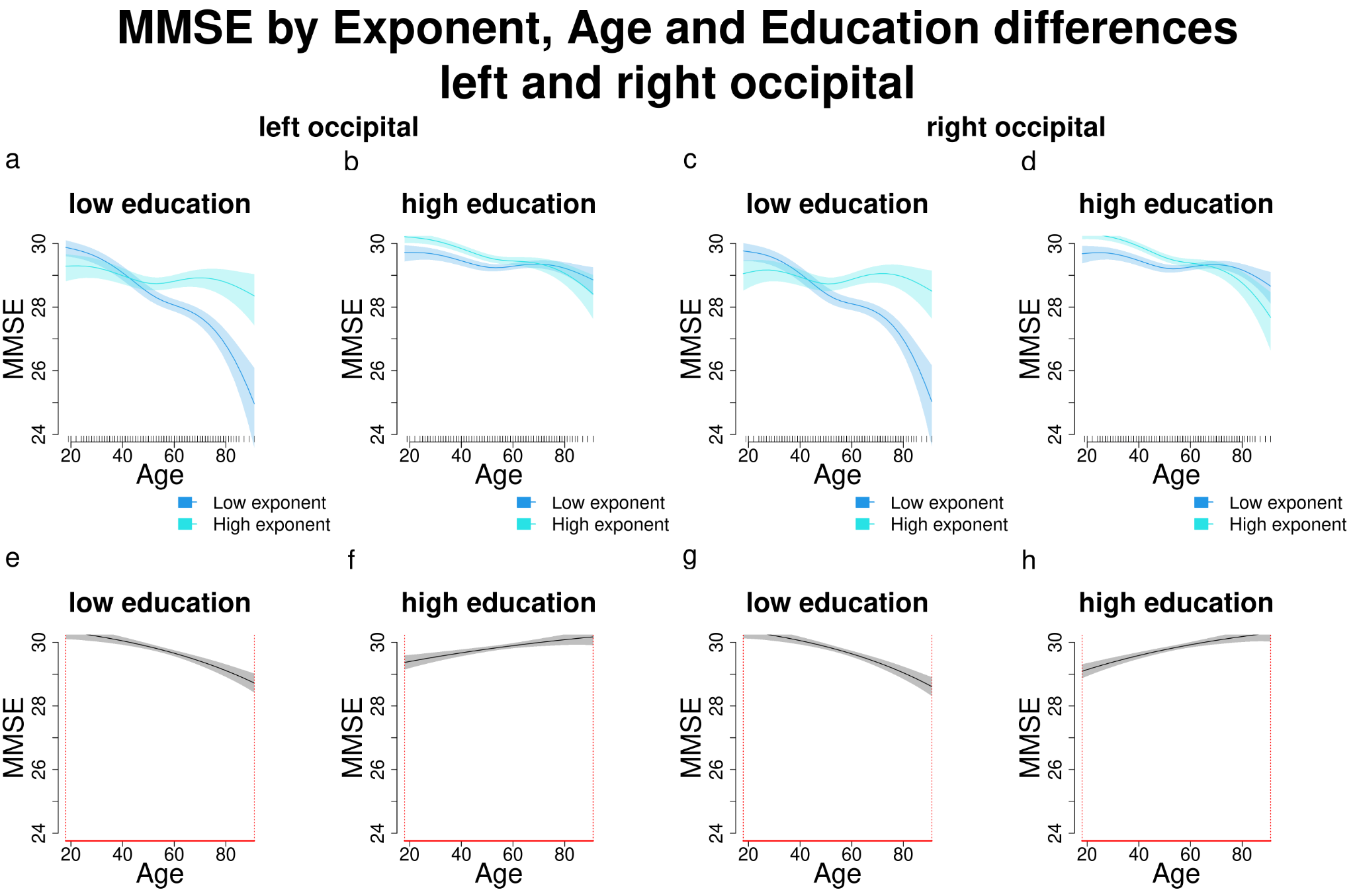


Figure S12. Comparisons and significant differences between MMSE scores of participants with high and low exponent, in relation with age and different education levels for the bilateral occipital region.


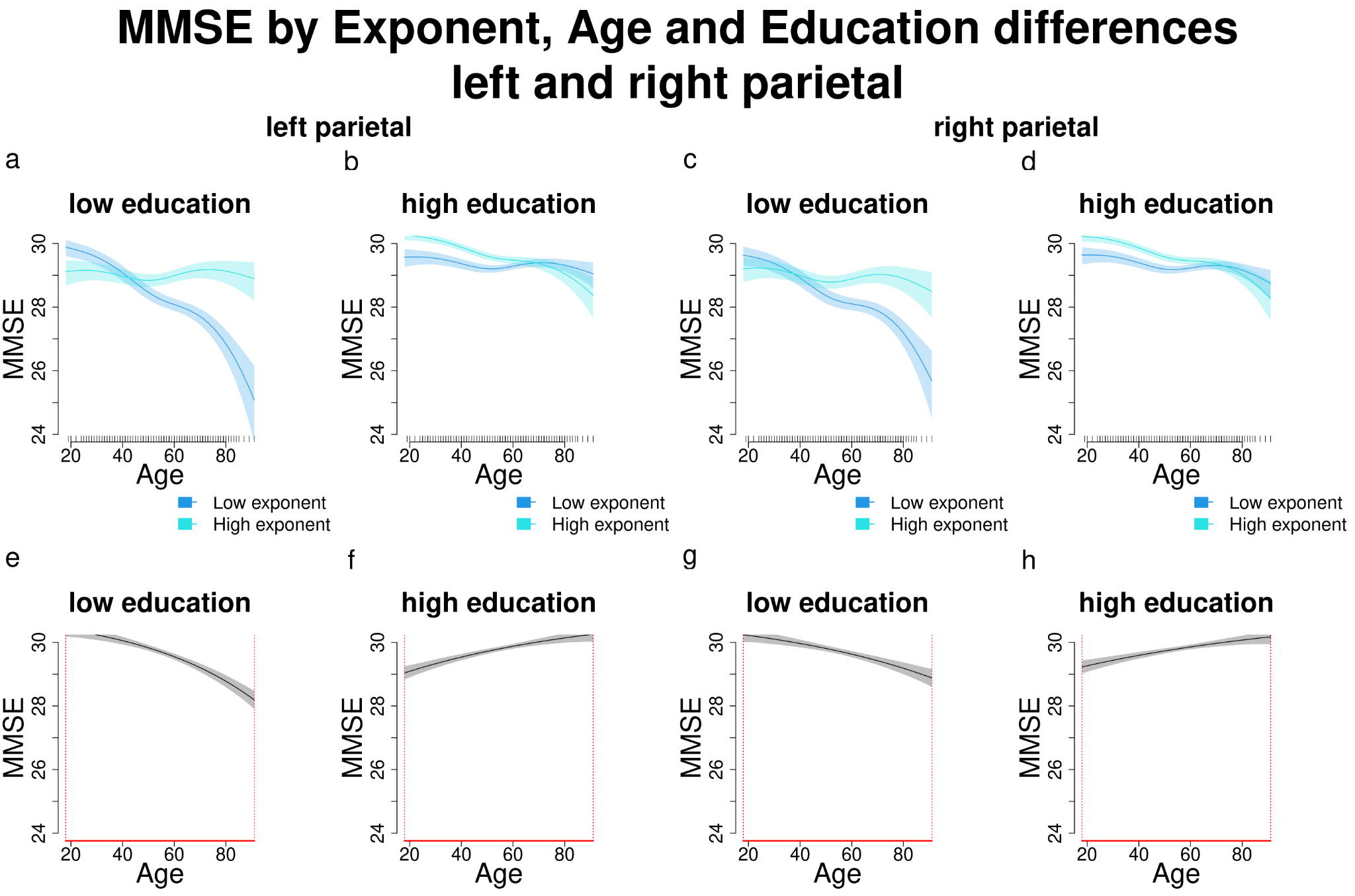


Figure S13. Comparisons and significant differences between MMSE scores of participants with high and low exponent, in relation with age and different education levels for the bilateral parietal regions.

## MMSE and Offset

Table S5. Significance of the smooth terms (interactions and main effects of age, education and offset) for the MMSE and offset models.

|  | ROI | deviance | EDF | F | p value | Sig |
| --- | --- | --- | --- | --- | --- | --- |
| s(Age) | CING_left | 21.288 | 3.643 | 11.833 | <0.001 | * |
| s(Educ) | CING_left | 21.288 | 2.297 | 12.317 | <0.001 | * |
| s(slope) | CING_left | 21.288 | 2.251 | 8.676 | <0.001 | * |
| ti(Age,Educ) | CING_left | 21.288 | 1.173 | 0.164 | 0.87 |  |
| ti(Age,slope) | CING_left | 21.288 | 2.106 | 1.14 | 0.351 |  |
| ti(Age,Educ,slope) | CING_left | 21.288 | 1.000 | 4.864 | 0.028 | * |
| s(Age) | CING_right | 21.657 | 3.902 | 9.453 | <0.001 | * |
| s(Educ) | CING_right | 21.657 | 2.375 | 10.58 | <0.001 | * |
| s(slope) | CING_right | 21.657 | 2.731 | 6.784 | <0.001 | * |
| ti(Age,Educ) | CING_right | 21.657 | 1.43 | 0.362 | 0.714 |  |
| ti(Age,slope) | CING_right | 21.657 | 2.084 | 0.87 | 0.371 |  |
| ti(Age,Educ,slope) | CING_right | 21.657 | 2.152 | 0.763 | 0.517 |  |
| s(Age) | HPC_left | 21.547 | 3.905 | 9.502 | <0.001 | * |
| s(Educ) | HPC_left | 21.547 | 1 | 26.071 | <0.001 | * |
| s(slope) | HPC_left | 21.547 | 1 | 9.858 | 0.002 | * |
| ti(Age,Educ) | HPC_left | 21.547 | 2.009 | 1.141 | 0.406 |  |
| ti(Age,slope) | HPC_left | 21.547 | 2.911 | 1.622 | 0.129 |  |
| ti(Age,Educ,slope) | HPC_left | 21.547 | 1 | 5.203 | 0.023 | * |
| s(Age) | HPC_right | 19.732 | 4.275 | 7.927 | <0.001 | * |
| s(Educ) | HPC_right | 19.732 | 1.826 | 13.978 | <0.001 | * |
| s(slope) | HPC_right | 19.732 | 1 | 10.835 | 0.001 | * |
| ti(Age,Educ) | HPC_right | 19.732 | 1.816 | 0.95 | 0.458 |  |
| ti(Age,slope) | HPC_right | 19.732 | 1.889 | 0.51 | 0.638 |  |
| ti(Age,Educ,slope) | HPC_right | 19.732 | 1.986 | 1.049 | 0.335 |  |
| s(Age) | OCC_left | 20.537 | 3.958 | 9.297 | <0.001 | * |
| s(Educ) | OCC_left | 20.537 | 2.162 | 11.885 | <0.001 | * |
| s(slope) | OCC_left | 20.537 | 1.558 | 6.074 | 0.002 | * |
| ti(Age,Educ) | OCC_left | 20.537 | 1.362 | 0.67 | 0.612 |  |
| ti(Age,slope) | OCC_left | 20.537 | 1.752 | 0.628 | 0.508 |  |
| ti(Age,Educ,slope) | OCC_left | 20.537 | 2.434 | 3.649 | 0.016 | * |
| s(Age) | OCC_right | 20.104 | 4.394 | 7.839 | <0.001 | * |
| s(Educ) | OCC_right | 20.104 | 2.151 | 10.85 | <0.001 | * |
| s(slope) | OCC_right | 20.104 | 1.129 | 8.117 | 0.002 | * |
| ti(Age,Educ) | OCC_right | 20.104 | 1.585 | 0.651 | 0.518 |  |
| ti(Age,slope) | OCC_right | 20.104 | 2.216 | 1.317 | 0.216 |  |
| ti(Age,Educ,slope) | OCC_right | 20.104 | 1 | 6.07 | 0.014 | * |
| s(Age) | PARIET_left | 21.383 | 4.09 | 11.166 | <0.001 | * |
| s(Educ) | PARIET_left | 21.383 | 1 | 22.791 | <0.001 | * |
| s(slope) | PARIET_left | 21.383 | 1.327 | 7.83 | 0.001 | * |
| ti(Age,Educ) | PARIET_left | 21.383 | 1 | 0.426 | 0.514 |  |
| ti(Age,slope) | PARIET_left | 21.383 | 2.168 | 1.005 | 0.273 |  |
| ti(Age,Educ,slope) | PARIET_left | 21.383 | 4.993 | 1.809 | 0.071 |  |
| s(Age) | PARIET_right | 20.014 | 4.044 | 10.815 | <0.001 | * |
| s(Educ) | PARIET_right | 20.014 | 2.697 | 8.31 | <0.001 | * |
| s(slope) | PARIET_right | 20.014 | 1.745 | 7.31 | 0.001 | * |
| ti(Age,Educ) | PARIET_right | 20.014 | 1 | 0.42 | 0.517 |  |
| ti(Age,slope) | PARIET_right | 20.014 | 1.982 | 0.809 | 0.436 |  |
| ti(Age,Educ,slope) | PARIET_right | 20.014 | 1 | 3.648 | 0.057 |  |
| s(Age) | TEMP_left | 21.56 | 3.898 | 9.957 | <0.001 | * |
| s(Educ) | TEMP_left | 21.56 | 1 | 25.01 | <0.001 | * |
| s(slope) | TEMP_left | 21.56 | 1 | 4.457 | 0.035 | * |
| ti(Age,Educ) | TEMP_left | 21.56 | 2.148 | 1.756 | 0.251 |  |
| ti(Age,slope) | TEMP_left | 21.56 | 3.996 | 2.121 | 0.051 |  |
| ti(Age,Educ,slope) | TEMP_left | 21.56 | 2.887 | 1.87 | 0.13 |  |
| s(Age) | TEMP_right | 20.877 | 4.287 | 7.813 | <0.001 | * |
| s(Educ) | TEMP_right | 20.877 | 2.609 | 9.911 | <0.001 | * |
| s(slope) | TEMP_right | 20.877 | 1 | 12.565 | <0.001 | * |
| ti(Age,Educ) | TEMP_right | 20.877 | 1.389 | 0.605 | 0.633 |  |
| ti(Age,slope) | TEMP_right | 20.877 | 1 | 0.192 | 0.662 |  |
| ti(Age,Educ,slope) | TEMP_right | 20.877 | 6.523 | 1.075 | 0.364 |  |

As for the exponent models, also for the offset the complex models showed a better fit than the baseline MMSE model in all ROIs.

### Complex models’ figures

Figures S14-S17 depict MMSE score changes according to age and education across different offset levels (results of the complex models’ interactions) for the bilateral cingulate, hippocampus, occipital and parietal ROIs. Darker blue shades indicate lower MMSE scores, while lighter green and yellow shades indicate higher MMSE scores. White areas indicate model estimates exceeding the upper MMSE score limit of 30.

Figures S18-S21 depict the comparisons and significant differences between MMSE scores of participants with high and low offset, in relation with age and different education levels for the bilateral cingulate, hippocampus, occipital and parietal ROIs. Upper panels show how different offset levels shape MMSE scores across different levels of education (high and low) and increasing age. Lower panels show significant differences in MMSE scores between participants with varying offset and education levels across age. In general, participants with low education and higher offsets have significantly higher MMSE scores than those with lower offsets starting approximately from age 40. Moreover, participants with high education and higher offsets have worse MMSE scores than those with lower offsets. In lower panels, age ranges when differences are significant are highlighted with a red line on the x axis (MMSE score differences are significant across the entire age window).


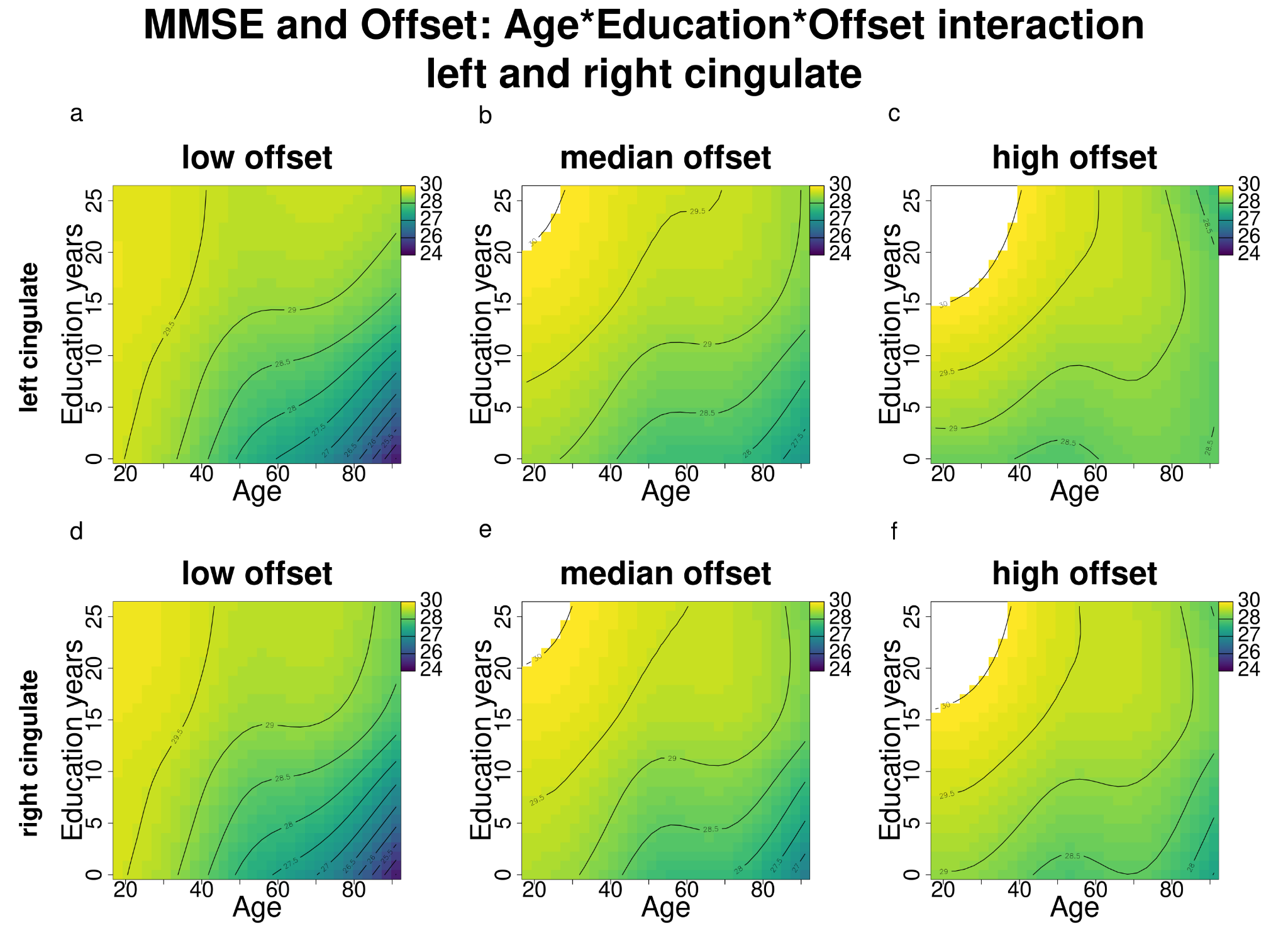


Figure S14. MMSE score changes according to age and education across different offset levels for the bilateral cingulate.


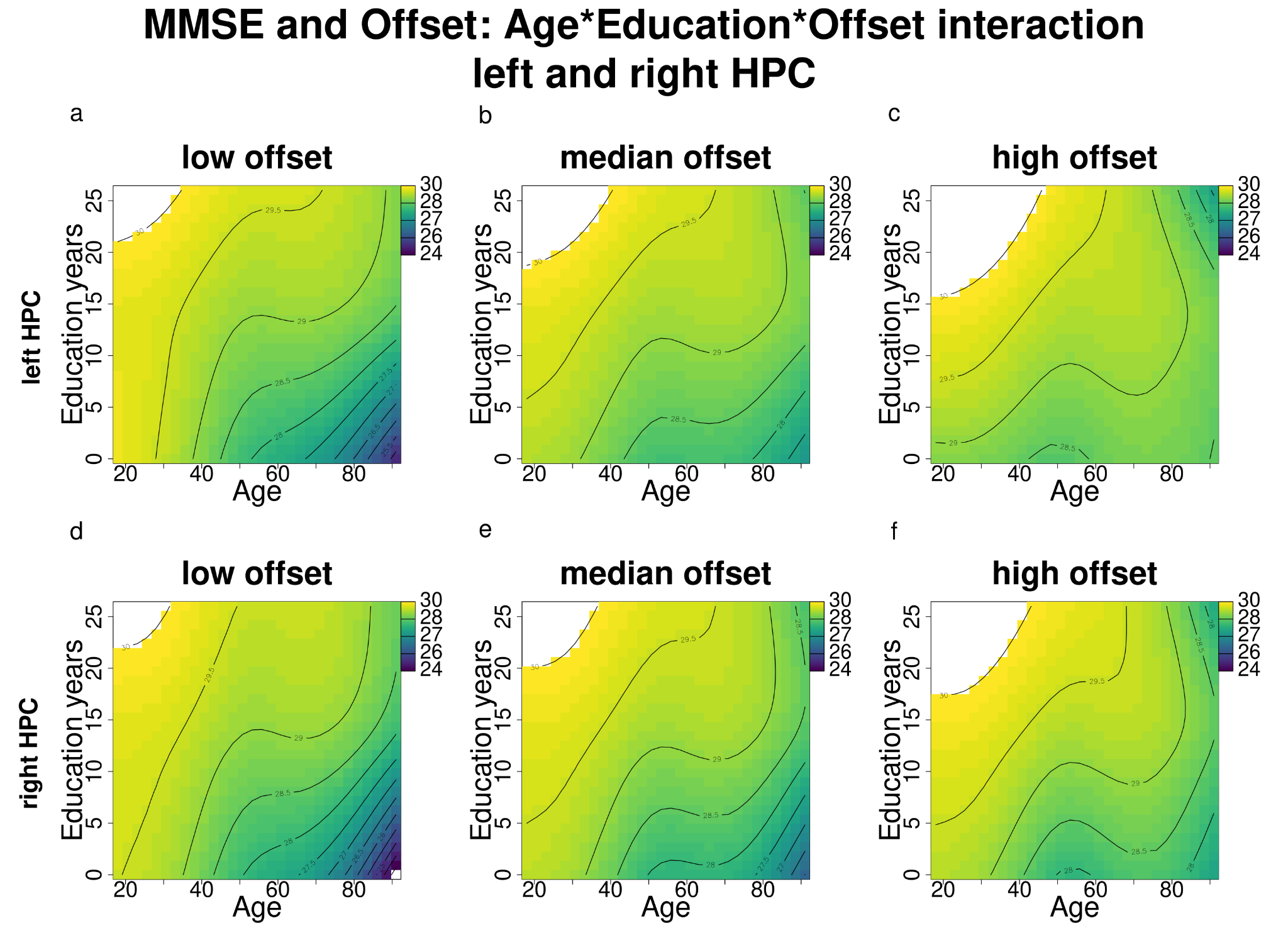


Figure S15. MMSE score changes according to age and education across different offset levels for the bilateral hippocampus.


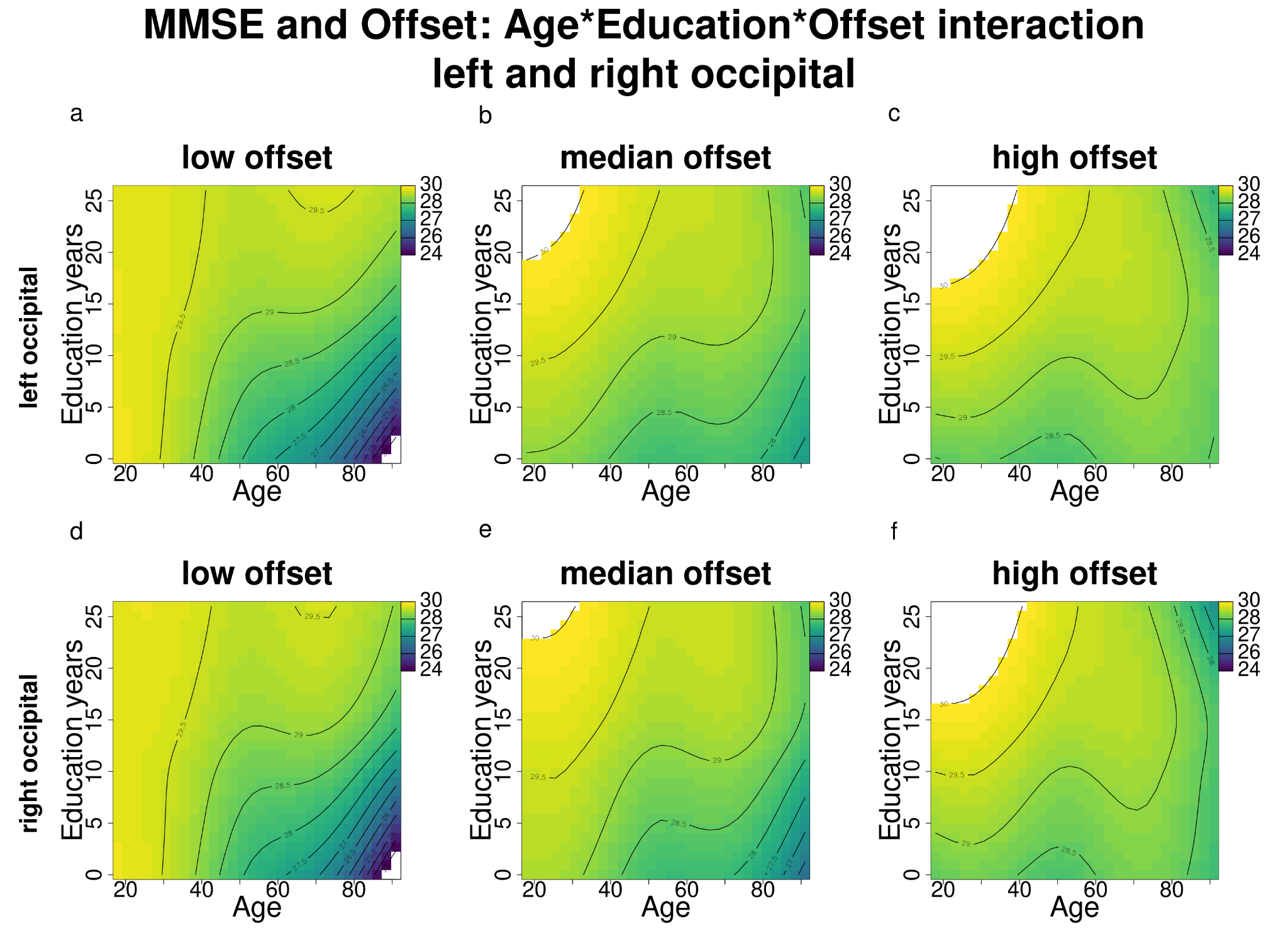


Figure S16. MMSE score changes according to age and education across different offset levels for the bilateral occipital region.


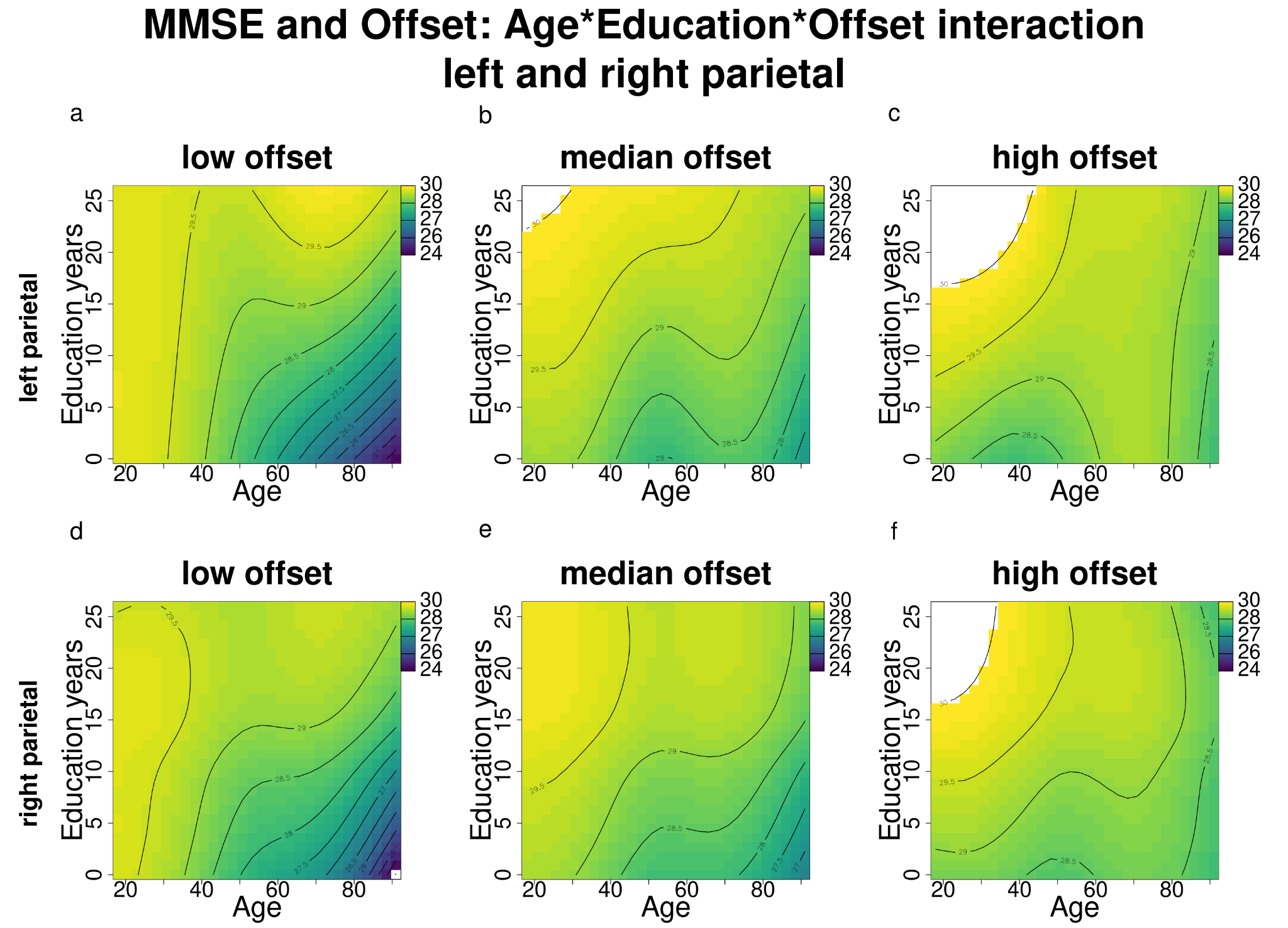


Figure S17. MMSE score changes according to age and education across different offset levels for the bilateral parietal regions.


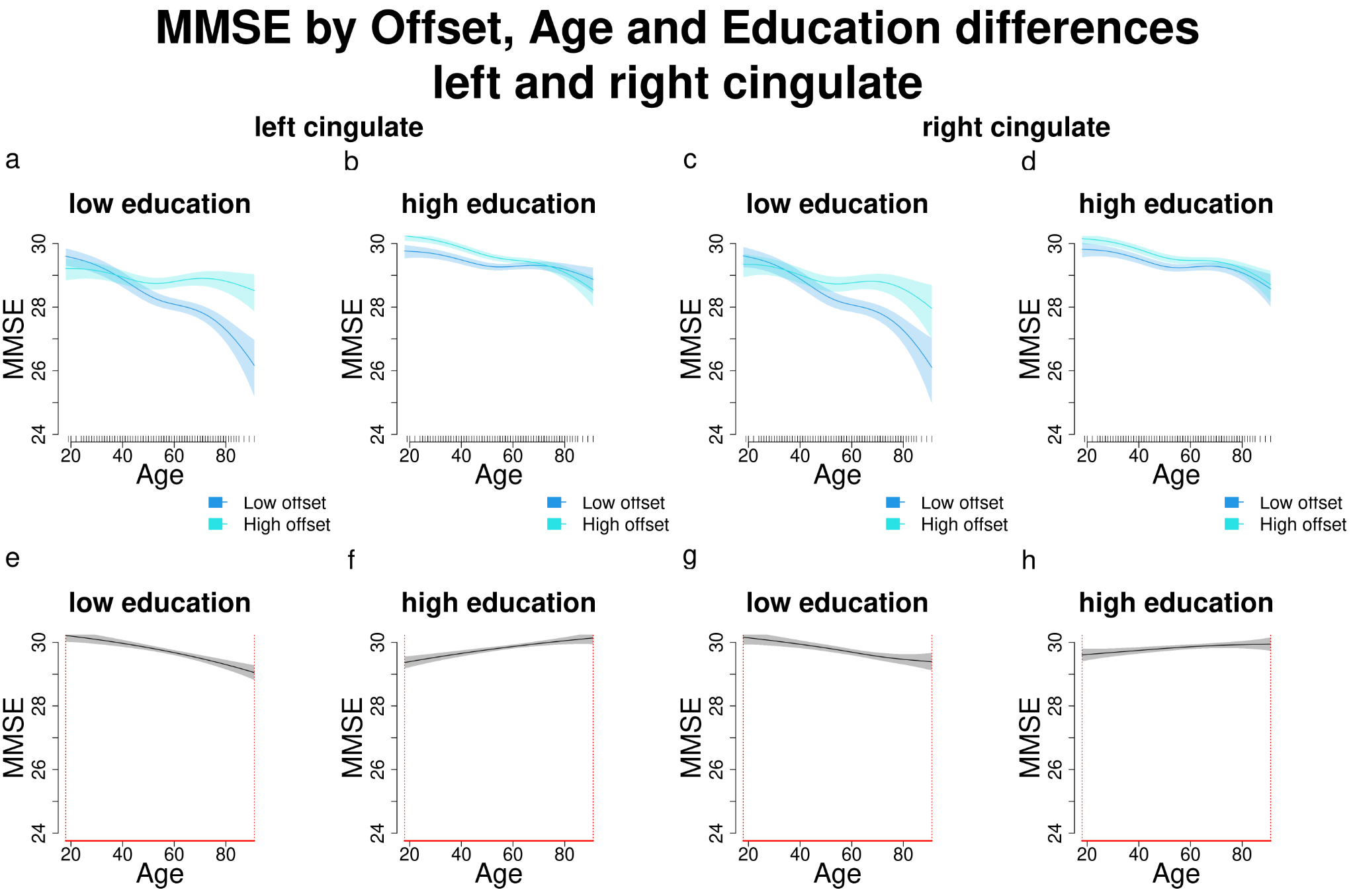


Figure S18. Comparisons and significant differences between MMSE scores of participants with high and low offsets, in relation with age and different education levels for the bilateral cingulate.


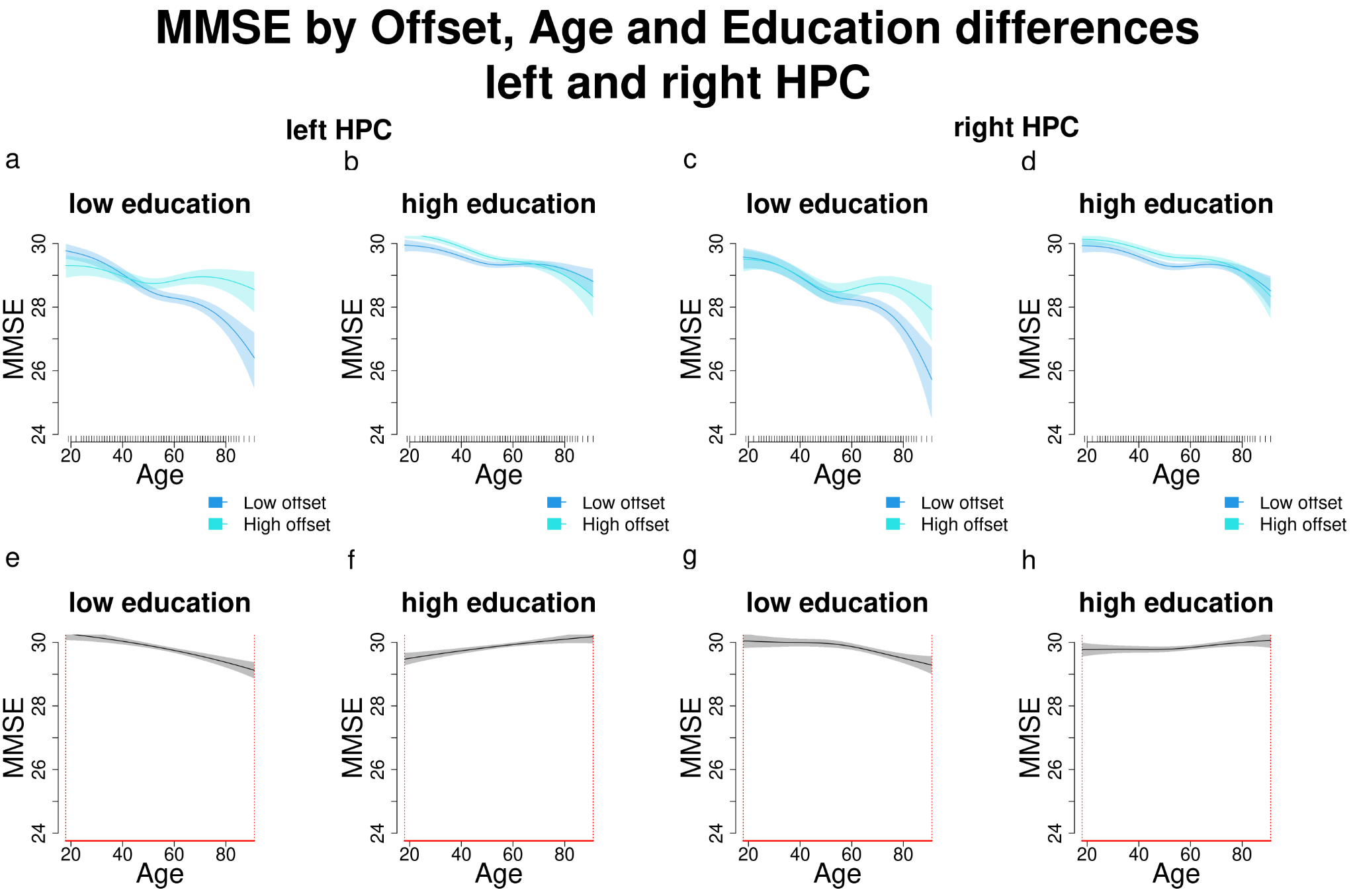


Figure S19. Comparisons and significant differences between MMSE scores of participants with high and low offsets, in relation with age and different education levels for the bilateral hippocampus.


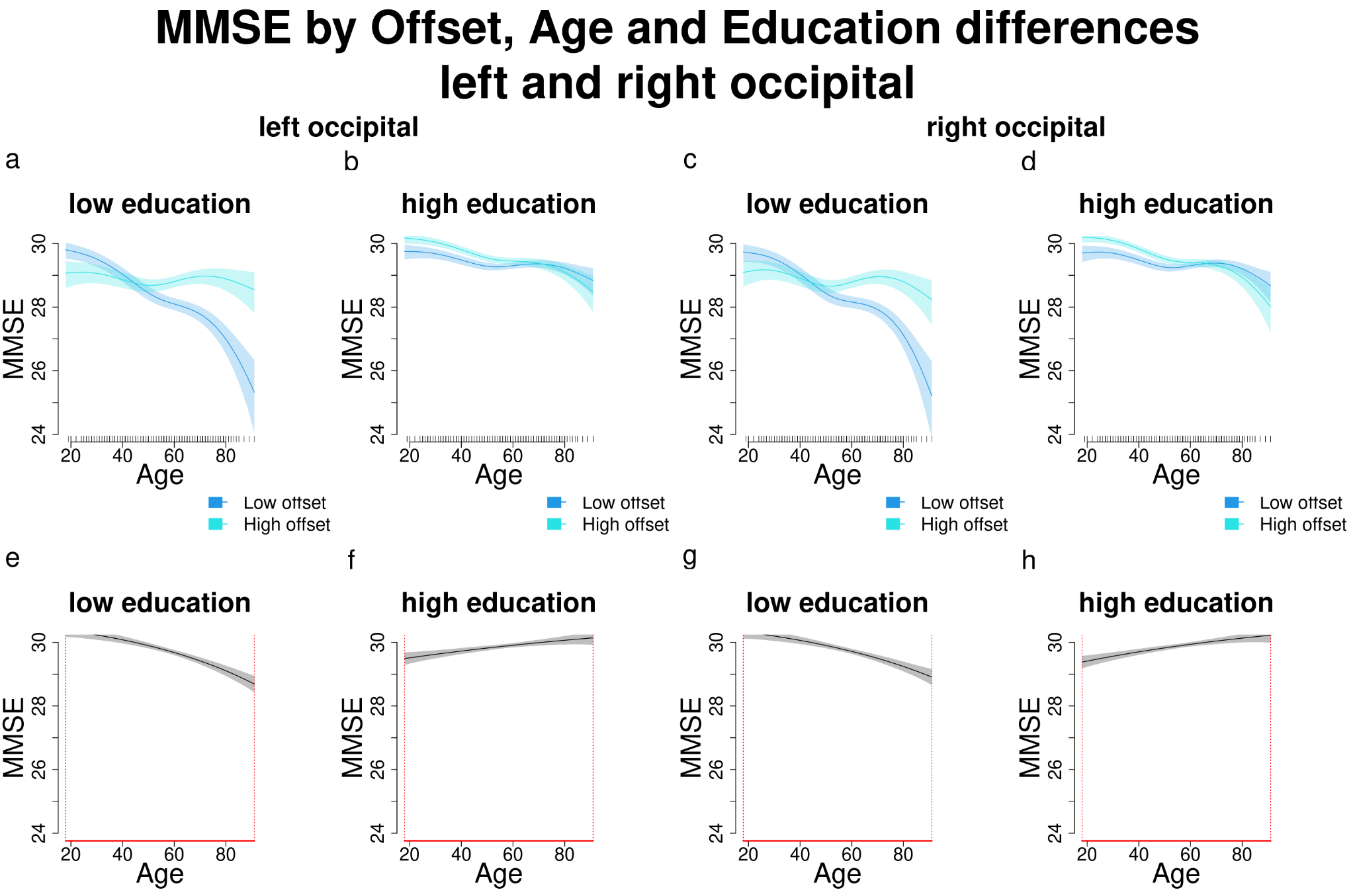


Figure S20. Comparisons and significant differences between MMSE scores of participants with high and low offsets, in relation with age and different education levels for the bilateral occipital region.


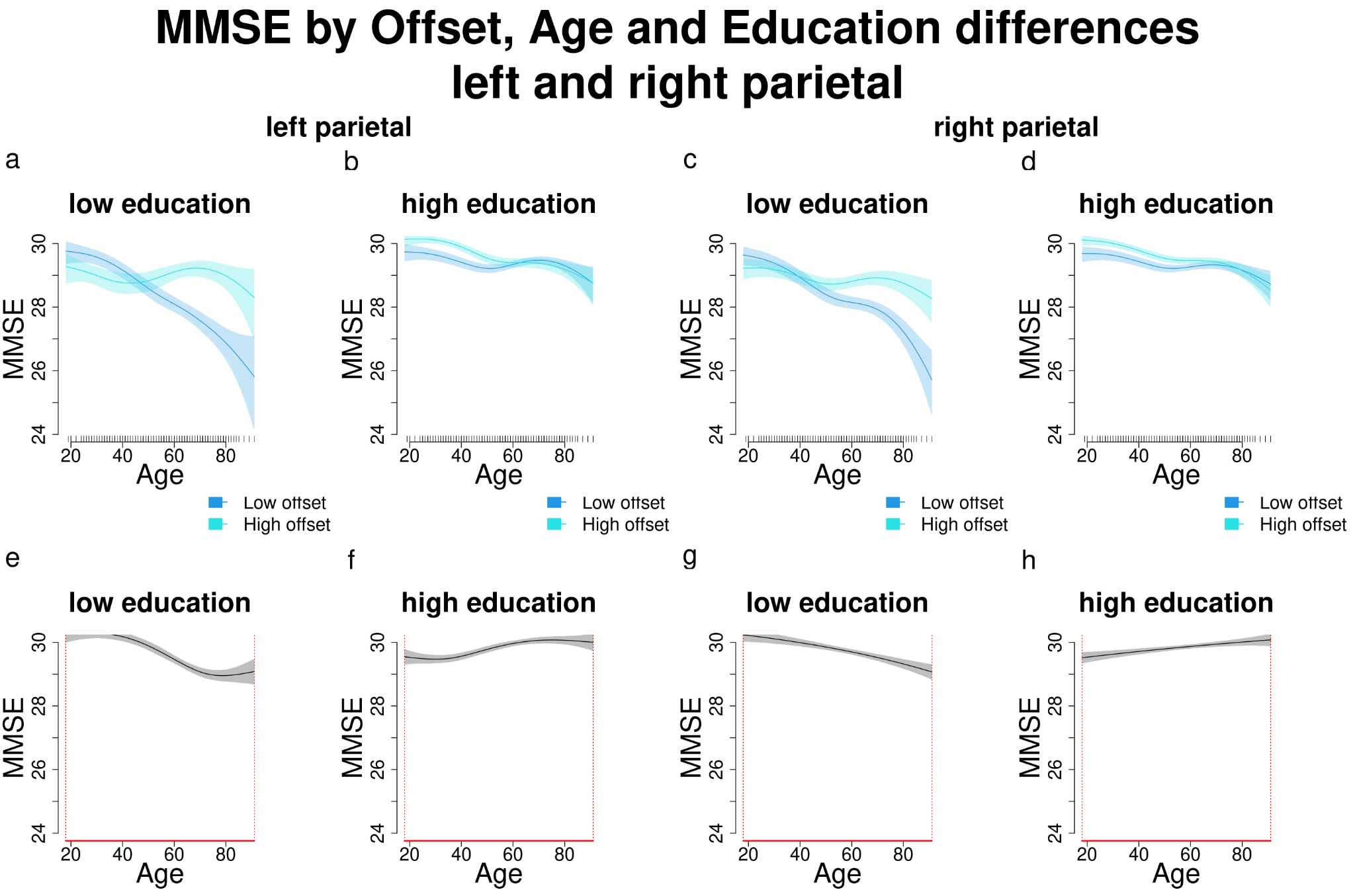


Figure S21. Comparisons and significant differences between MMSE scores of participants with high and low offsets, in relation with age and different education levels for the bilateral parietal region.

## Exponent and offset correlation

Table S6. Spearman’s correlation coefficients between exponent and offset for each ROI.

| ROI | r | p | Sig |
| --- | --- | --- | --- |
| CING_left | 0.957 | <0.001 | * |
| CING_right | 0.964 | <0.001 | * |
| HPC_left | 0.950 | <0.001 | * |
| HPC_right | 0.937 | <0.001 | * |
| OCC_left | 0.962 | <0.001 | * |
| OCC_right | 0.960 | <0.001 | * |
| PARIET_left | 0.966 | <0.001 | * |
| PARIET_right | 0.970 | <0.001 | * |
| TEMP_left | 0.967 | <0.001 | * |
| TEMP_right | 0.961 | <0.001 | * |
