# Supplementary material for: Education shapes the link between EEG aperiodic components and cognitive aging
Source: PLoS One. 2026 Mar 9;21(3):e0328318. doi: 10.1371/journal.pone.0328318 (PMC12970889; doi:10.1371/journal.pone.0328318)
Supplement: Attachment — Submitted filename: Response to Reviewers.docx [file pone.0328318.s003.docx]

Review Comments to the Author

Reviewer #1: I have read the response to the reviewers and the update to the manuscript. Broadly, the authors have responded to main points and made edits to the manuscript that address several of the original comments. However, in doing so I find there to be some issues with how updates to the manuscript are phrased and presented. For example, the authors seem to mis-represent some points about the relationship between the exponent and the offset, as if there are two competing viewpoints about the offset (that it is correlated with the exponent vs it being a separate phenomenon). These are not opposing views - the correlation of the offset and exponent is a finding, not an interpretation, based on the offset being impacted by any non-zero frequency rotation point change in exponent. It is also true that the offset can change and be interpreted separately from the exponent, if one analyzes for this situation. Without demonstrating this, however, the offset cannot be interpreted separately from the offset. Related to this, some of the phrasing is poor. For example, it is not clear what “exponent and offset are highly correlated due to their shared influence on the power spectrum” is actually supposed to mean.

The offset and the exponent do not have shared influence on the PSD, they are measures of the PSD. These descriptions do not seem to reflect a clear perspective and interpretation of the phenomena under study. It is also not clear why authors do not report the any correlation measures between offset and exponent in this dataset to speak to this topic in a more empirical sense. Some of the other edits are also not particularly precise in relation to the literature - for example, I am not clear why the Salvatore paper, which is largely about pharmacological manipulations, is cited in the introduction as if the main comparison relates to sleep vs wakefulness. Overall, I think the authors need to ensure they revisit the related literature and their framing of it to make sure their descriptions of features of aperiodic activity are clear and well-described.

***Answer:*** Thank you for raising these points, that helped us improve our phrasing and clarify our point of view. We will answer here point by point.

- **The authors seem to mis-represent some points about the relationship between the exponent and the offset, as if there are two competing viewpoints about the offset (that it is correlated with the exponent vs it being a separate phenomenon) etc.**
  - We chose to run the same analyses both exponent and offset a) because they refer to different measures of the PSD, and b) to be consistent with the literature that considers these two aperiodic components as different but related aspects of the background neural activity. For these reasons, throughout the paper we explicitly acknowledge that we are not trying to interpret the offset as separate from the exponent without sufficient support from our analyses and findings (e.g., lines 47-61 “... exponent-offset correlations are not competing with interpretations of these components as having separate neural generators, but rather expected properties of the model…”; lines 148-157: “we quantified the aperiodic exponent and offset separately, but we interpret them jointly, acknowledging their strong interdependence due to the mathematical property of power spectrum rotation… We therefore refrain from assigning parameter-specific physiological mechanisms and instead frame our results in terms of general aperiodic dynamics, consistent with multiple plausible biophysical interpretations…”; lines 624-648: “... offset values cannot be interpreted as reflecting a distinct physiological process unless independent variation with respect to the exponent can be empirically demonstrated…”; lines 690-696: “... because exponent and offset share substantial variance due to rotation of the power spectrum, the offset cannot be interpreted as reflecting a distinct physiological mechanism unless independent variation is demonstrated…”. However, we also discuss the possible neurophysiological meaning of exponent and offset in line with the existing literature that does not interpret these two aperiodic components as completely overlapping.
- **It is also not clear why authors do not report the any correlation measures between offset and exponent in this dataset to speak to this topic in a more empirical sense.**
  - We computed correlation measures between exponent and offset (now reported in the Supplementary Materials) supporting their consideration that our findings are driven by the amount of shared variance between the two aperiodic components (lines 579-584; line 626). This additional finding does not alter the paper’s conclusions but further supports the idea that exponent and offset are closely related and are not easily distinguishable.
- **Some of the other edits are also not particularly precise in relation to the literature - for example, I am not clear why the Salvatore paper, which is largely about pharmacological manipulations, is cited in the introduction as if the main comparison relates to sleep vs wakefulness.**
  - We rephrased the context in which the Salvatore et al. paper is cited (lines 79), so that now it is clearer that it refers to pharmacological manipulations.

Reviewer #2: I have carefully reviewed the authors’ revisions and responses to my comments. Overall, I am satisfied with the changes, which have substantially improved the clarity and interpretability of the manuscript. I have only a few remaining minor recommendations: (i) add a reference to support the MMSE statement on line 162 (“generally, a score of 24 or higher indicates normal cognitive functioning”), (ii) label the x-axis in the education histograms with units (“years”), including in the supplementary figures, and (iii) explicitly acknowledge the lack of information on the timing/sequence of data collection as a limitation. With these minor revisions addressed, I am happy to recommend the manuscript for publication.

***Answer:*** All points have been addressed in the main text (point i, line 174; point iii, lines 190-191 and 771-772) and in the Supplementary Materials (point ii). Thank you for recommending the manuscript for publication.
